# Synthesis and Hemostatic Activity of New Amide Derivatives

**DOI:** 10.3390/molecules27072271

**Published:** 2022-03-31

**Authors:** Lukasz Banach, Lukasz Janczewski, Jakub Kajdanek, Katarzyna Milowska, Joanna Kolodziejczyk-Czepas, Grzegorz Galita, Wioletta Rozpedek-Kaminska, Ewa Kucharska, Ireneusz Majsterek, Beata Kolesinska

**Affiliations:** 1 Faculty of Chemistry, Institute of Organic Chemistry, Lodz University of Technology, Zeromskiego 116, 90-924 Lodz, Poland; lukasz.banach@dokt.p.lodz.pl (L.B.); lukasz.janczewski@p.lodz.pl (L.J.); 2 Department of General Biophysics, Faculty of Biology and Environmental Protection, University of Lodz, Pomorska 141/143, 90-236 Lodz, Poland; jakub.kajdanek@edu.uni.lodz.pl (J.K.); katarzyna.milowska@biol.uni.lodz.pl (K.M.); 3 Department of General Biochemistry, Faculty of Biology and Environmental Protection, University of Lodz, Pomorska 141/143, 90-236 Lodz, Poland; joanna.kolodziejczyk@biol.uni.lodz.pl; 4 Department of Clinical Chemistry and Biochemistry, Medical University of Lodz, 90-419 Lodz, Poland; grzegorz.galita@umed.lodz.pl (G.G.); wioletta.rozpedek@umed.lodz.pl (W.R.-K.); ireneusz.majsterek@umed.lodz.pl (I.M.); 5 Department of Gerontology, Geriatrics and Social Work, Jesuit University Ignatianum in Cracow, Kopernika 26, 31-501 Krakow, Poland; ewa.kucharska@ignatianum.edu.pl

**Keywords:** peptides, tranexamic acid, aminocaproic acid, 4-(aminomethyl)benzoic acid, antifibrinolytic agents, hemostasis, lysine analogs

## Abstract

Eight dipeptides containing antifibrinolytic agents (tranexamic acid, aminocaproic acid, 4-(aminomethyl)benzoic acid, and glycine—natural amino acids) were synthesized in a three-step process with good or very good yields. DMT/NMM/TsO^−^ (4-(4,6-dimethoxy-1,3,5-triazin-2-yl)-4-methylmorpholinium toluene-4-sulfonate) was used as a coupling reagent. Hemolysis tests were used to study the effects of the dipeptides on blood components. Blood plasma clotting tests were used to examine their effects on thrombin time (TT), prothrombin time (PT), and the activated partial thromboplastin time (aPTT). The level of hemolysis did not exceed 1%. In clotting tests, TT, PT, and aPTT did not differentiate any of the compounds. The prothrombin times for all amides **1**–**8** were similar. The obtained results in the presence of amides **1**–**4** and **8** were slightly lower than for the other compounds and the positive control, and they were similar to the results obtained for TA. In the case of amide **3**, a significantly decreased aPTT was observed. The aPTTs observed for plasma treated with amide **3** and TA were comparable. In the case of amide **6** and **8**, TT values significantly lower than for the other compounds were found. The clot formation and fibrinolysis (CFF) assay was used to assess the influence of the dipeptides on the blood plasma coagulation cascade and the fibrinolytic efficiency of the blood plasma. In the clot formation and fibrinolysis assay, amides **5** and **7** were among the most active compounds. The cytotoxicity and genotoxicity of the synthesized dipeptides were evaluated on the monocyte/macrophage peripheral blood cell line. The dipeptides did not cause hemolysis at any concentrations. They exhibited no significant cytotoxic effect on SC cells and did not induce significant DNA damage.

## 1. Introduction

**Figure 1:**
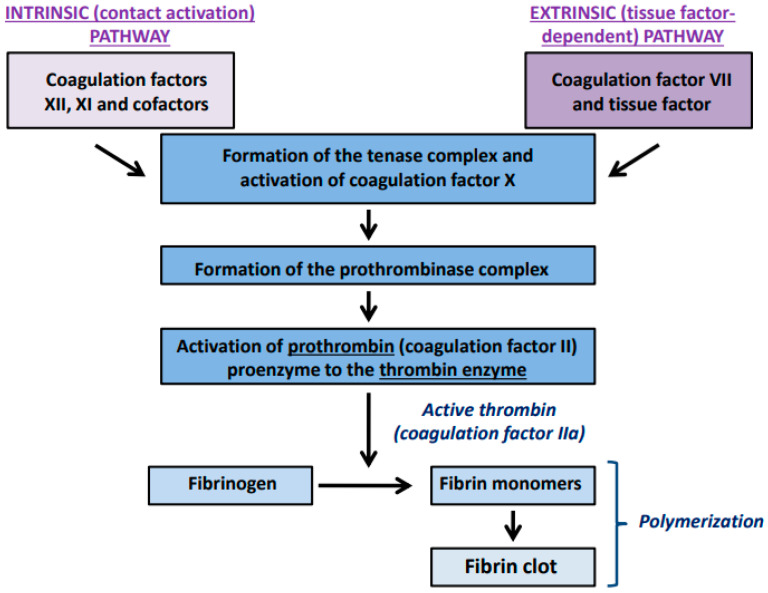
Key steps in the blood plasma coagulation cascade. The extrinsic pathway is activated by the tissue factor, which is abundantly expressed in the subendothelial tissue but becomes available to blood plasma proteins after vascular injury. Induction of the intrinsic coagulation pathway requires activation of the coagulation factor XII and the presence of a high-molecular (HMW) kininogen and prekallikrein. Independently on the starting point, both pathways trigger the tenase and prothrombinase enzymatic complexes, leading to the generation of the thrombin enzyme. The thrombin-catalyzed removal of short peptides from the fibrinogen molecule activates fibrinogen polymerization and the formation of fibrin monomers and polymers. This complex cascade of reactions results in the conversion of soluble fibrinogen into an insoluble fibrin clot.

Hemostasis involves a complex network of interactions between blood components and the blood vessel wall, which is responsible for inhibiting bleeding after injuries to the blood vessels and for maintaining the fluidity of circulating blood. It requires an appropriate balance between the coagulation cascade, which leads to the formation of fibrin clot, and the fibrinolytic system, which removes fibrin clots [1]. The critical point in the blood coagulation is a transformation of the soluble plasma protein, fibrinogen, into a fibrin clot preventing the blood leakage after disruption of a blood vessel wall (Figure 1) [2].

**Figure 2:**
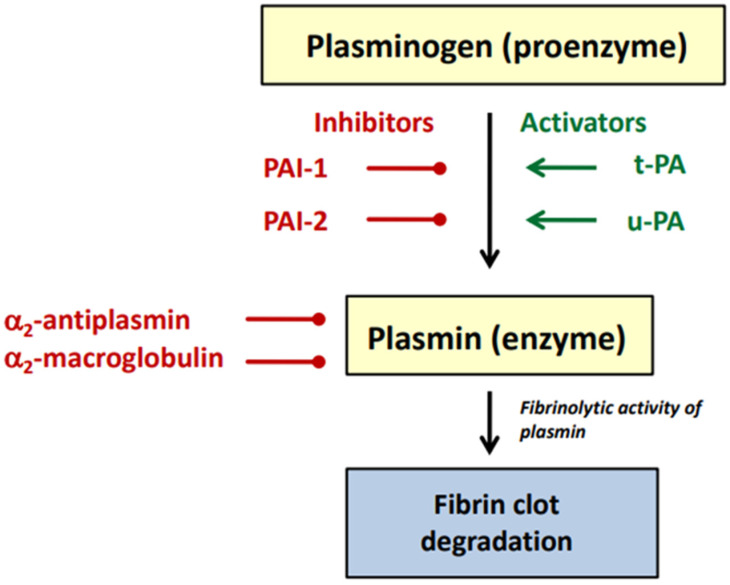
Main components of the fibrinolytic system (description in the text).

The main physiological role of the fibrinolytic system is to remove intravascular fibrin deposits and restore blood flow. Fibrinolysis is initiated by plasminogen activators, which convert the proenzyme plasminogen into the active serine protease-plasmin. Analogously to the coagulation cascade, activation of the fibrinolytic system may also be associated with the activity of components of the intrinsic pathway (prekallikrein, kininogen, and factor XII). However, an essential role in the stimulation of fibrinolytic activity plays the proteolytic activation of plasminogen to plasmin by the tissue-type plasminogen activator (t-PA) and the urokinase-type plasminogen activator (u-PA). The main activator of intravascular fibrinolysis is t-PA. The u-PA activator is involved in extravascular proteolysis, including tissue remodeling and repair. The fibrinolytic system can be inhibited on two levels. The first level is the plasminogen activation process, which is controlled by plasminogen activator inhibitors (PAIs). The main regulator of plasmin generation is the plasminogen activator inhibitor 1 (PAI-1), which is capable of inhibiting both t-PA and u-PA. Plasminogen activator inhibitor 2 (PAI-2) is of minor importance. It selectively inactivates u-PA. The second level of the fibrinolytic system regulation is the inactivation of plasmin, which is mediated by α_2_-antiplasmina and α_2_-macroglobulin. The main components of the fibrinolytic system are presented in Figure 2.

**Figure 3:**
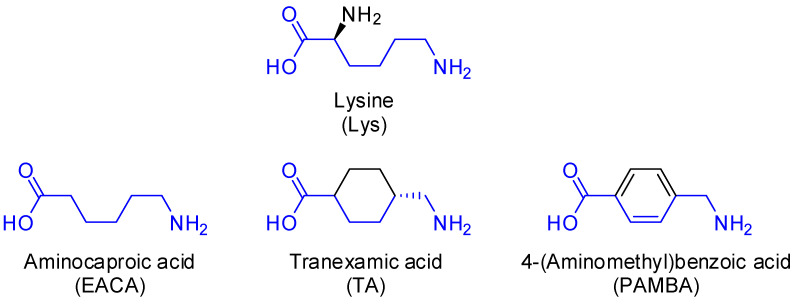
Structure of lysine and its synthetic analogs: aminocaproic acid (EACA), tranexamic acid (TA), and 4-(aminomethyl)benzoic acid (PAMBA).

Due to the increased risk of thromboembolic complications during many civilization diseases such as atherosclerosis, obesity, metabolic syndrome, and cancer, current therapeutic strategies have been mostly focused on anticoagulant drugs. However, disorders of fibrinolytic activity of blood plasma are also a serious medicinal challenge, especially in the context of the risk of uncontrolled bleedings during different surgical procedures. The number of fibrinolysis modulators available to patients is limited. Currently used antifibrinolytic agents include aprotinin—a naturally occurring competitive inhibitor of serine proteinases as well as synthetic lysine analogs such as tranexamic acid, aminocaproic acid, and 4-(aminomethyl)benzoic acid (Figure 3) [2]. Lysine itself displays weak antifibrinolytic activity.

The molecular mechanism of action by the main clinically used antifibrinolytic drugs belonging to the group of lysine analogs is the blockage of lysine-binding sites (LBS) in the plasminogen molecule. Lysine-binding sites are responsible for interactions between plasminogen and the fibrin surface, which is essential for its t-PA-mediated activation of plasmin. As a consequence, the blockage of LBS inhibits fibrin degradation. Tranexamic acid binds with plasminogen 6- to 10-fold more than aminocaproic acid in fibrinolytic test systems [3]. Synthetic lysine analogs [2] (tranexamic acid and aminocaproic acid) almost completely block the binding of plasminogen to fibrin. 

Tranexamic acid (*trans*-4-(aminomethyl)cyclohexane carboxylic acid, TA) is a synthetic derivative of lysine [1,3,4,5,6,7,8]. It is an antifibrinolytic agent that blocks LBS in plasminogen. For local fibrinolysis, the recommended dosage is between 500 mg and 1 g administered by slow intravenous injection three times daily or between 1 and 1.5 g administered orally two to three times daily. For general fibrinolysis, a single dose of 1 g or 10 mg/kg administered by slow intravenous injection is recommended [5]. The indications for which TA is approved vary in different countries. The range of indications is limited in some (e.g., the US [9]) and broad in others (e.g., Japan [10] and the UK [11]). Tranexamic acid is used in cardiac surgery [12,13,14,15,16,17,18,19], in orthopedic surgery [20,21,22], in spinal and cranial surgery [23], in hepatic surgery [24], in prostate surgery [25], in gynecology for heavy menstrual bleeding [26,27], in postpartum hemorrhage [28,29], in acute upper gastrointestinal bleeding [30,31], and in oral surgery [32]. After oral administration of TA, gastrointestinal side effects have been reported, including nausea, diarrhea, and abdominal cramping. These adverse effects have been not reported with intravenous administration. Rapid intravenous administration may cause hypotension [2].

Similar to TA, aminocaproic acid (ε-aminocaproic acid, 6-aminocaproic acid, 6-aminohexanoic acid, EACA) is a competitive inhibitor of plasminogen activation. The recommended dose of EACA is 150 mg/kg as an intravenous bolus before surgery, which is followed by an infusion of 15 mg/kg/h during the operation [33]. It is used in cardiac surgery [34,35] and orthotopic liver transplantation [36]. The most common side effect of rapid intravenous administration of EACA is hypotension. Occasionally, during longer-term administration, patients can suffer from rashes, nausea, vomiting, weakness, retrograde ejaculation, myopathy, and rhabdomyolysis [37].

4-(Aminomethyl)benzoic acid (para-(aminomethyl)benzoic acid, PAMBA) is another antifibrinolytic agent but a weak competitive inhibitor of plasminogen activation. It is a type II antifibrinolytic agent that is used for the treatment of fibrotic skin problems, such as Peyronie’s disease [38,39].

**Figure 4:**
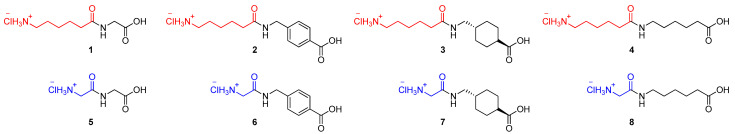
Structures of dipeptides **1**–**8** with expected hemostatic activities.

This study aimed to synthesize eight new dipeptides **1**–**8** derivatives containing tranexamic acid, aminocaproic acid, and 4-(aminomethyl)benzoic acid (synthetic analogs of lysine) composed exclusively of unnatural amino acids or their combination with glycine (Figure 4). As a result of the known hemostatic properties of synthetic lysine analogs, the designed derivatives were expected to have antifibrinolytic properties. Therefore, we evaluated the effects of the synthesized dipeptide derivatives on the hemostatic activity of blood plasma and their activity toward blood cells.

## 2. Results and Discussion

### 2.1. Chemistry

**Figure 5:**
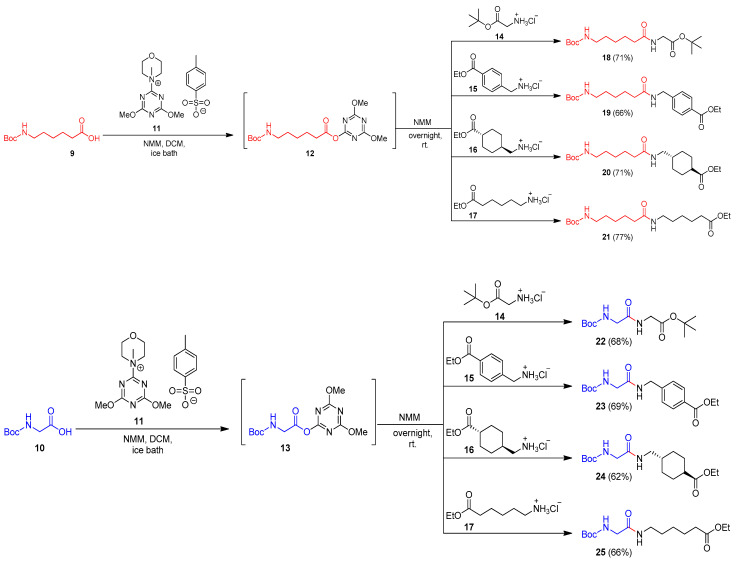
Synthesis of *N*-Boc amides **18**–**25**.

The first step was the synthesis of *N*-Boc alkyl (*tert*-butyl or ethyl) esters of amides **18**–**25**. The synthesis was carried out in solution (DCM as a solvent). The *N*-protected unnatural amino acid 6-(*N*-Boc-amino)caproic acid (**9**) and the *N*-protected natural amino acid *N*-Boc-glycine (**10**) were used as the main carboxylic substrates. In the presence of 4-(4,6-dimethoxy-1,3,5-triazin-2-yl)-4-methylmorpholiniumtoluene-4-sufonate (DMT/NMM/TsO^−^, **11**) [40,41] as a coupling reagent, they were transformed into intermediate active esters (**12** and **13**, respectively). This step was performed in an ice bath in the presence of *N*-methylmorpholine (NMM). The formation of active ester **12** was controlled by TLC. The preparation of active ester **13** took only 30 min because with the passage of time, the formed intermediate product **13** was decomposed. After that, four different hydrochlorides were used: one natural hydrochloride, glycine *tert*-butyl (**14**), and three unnatural hydrochlorides, ethyl ester of 4-(aminomethyl)benzoic acid (**15**), ethyl ester of *trans*-4-(aminomethyl)cyclohexanecarboxylic acid (**16**), and ethyl ester of 6-aminocaproic acid **17**). *N*-Boc amides **18**–**25** were isolated after flash chromatography (hexane/ethyl acetate 1:1) or crystallization (hexane) with satisfactory yields (62–77%) and high purity (Figure 5).

**Figure 6:**
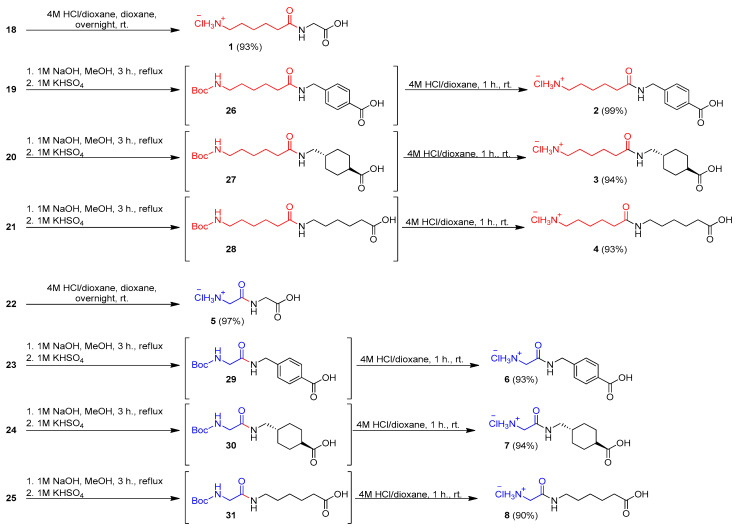
Synthesis of hydrochlorides of amides **1**–**8**.

The second and third steps were hydrolysis of the ester group and deprotection of the Boc group. Hydrolysis of compounds **19**–**21** and **23**–**25** containing ethyl ester was carried out in the presence of 1 M NaOH in methanol for 3 h in reflux. Then, the solution was acidified to pH 2–3 using 1 M KHSO_4_, and crude products **26**–**31**, after evaporation of the solvent, and without other purification, were used in the next step. Deprotection of Boc-derivatives **26**–**28** and **29**–**31** was performed under acid conditions using 4 M HCl in dioxane. The final amides **2**–**4** and **6**–**8** were isolated after crystallization with diethyl ether, with more than 90% yield (Figure 6).

In the case of amides **18** and **22** with a *tert*-butyl moiety both in the ester group and in the Boc group, deprotection and hydrolysis were performed in one step using 4 M HCl in dioxane. Final hydrochlorides of amides **1** and **5** were isolated after crystallization with Et_2_O with almost quantitative yields (Figure 6).

### 2.2. Biological Activity

Due to the prevalence to prothrombotic complications in many diseases, numerous studies devoted to substances with anticoagulant activity have been executed [42,43]. In contrast to advances in the antithrombotic therapy development, research on the modulation of fibrinolytic proteins is less advanced. Currently available antifibrinolytic therapies have several limitations [44]. Aprotinin, a Kunitz-type serine protease inhibitor, is the only antifibrinolytic drug approved for the direct inhibition of plasmin activity. However, its clinical use is limited by several serious side effects. Research on new, safer plasmin inhibitors has not yet provided satisfactory results. Studies on the interactions between plasmin active sites and potential inhibitors (including peptide-type substances) are only at the preliminary stages of in vitro tests or in silico prediction [45]. Another trend in recent research on new natural and synthetic substances with antifibrinolytic agents is the allosteric modulation of plasmin activity [46]. For example, synthetic sulfated small molecules have been found to have plasmin-inhibitory activity in vitro, in a study in which 55 compounds were screened. A pentasulfated flavonoid–quinazolinone dimer was the most effective plasmin inhibitor (IC_50_ = 45 μM) [47]. Our previous studies showed that natural compounds belonging to the bufadienolide group can also act as uncompetitive inhibitors of plasmin in vitro [48].

In the present study, we synthesized new amide derivatives containing well-known antifibrinolytic compounds. We conducted hemolytic as well as genotoxic tests on the new amide derivatives and evaluated their cellular safety and antifibrinolytic potential in vitro.

#### 2.2.1. Hemolysis

**Figure 7:**
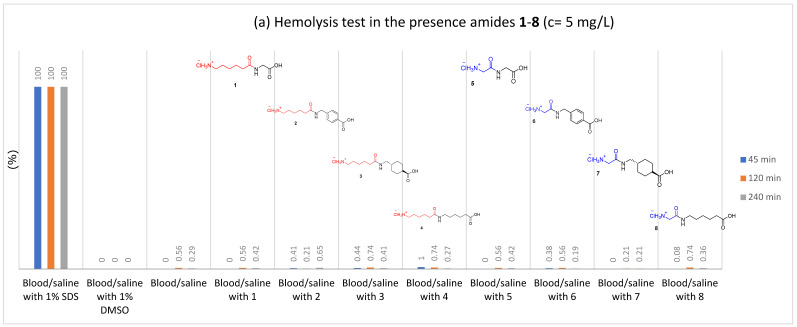

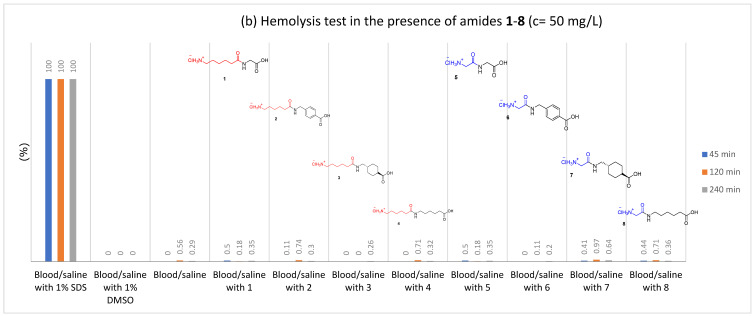

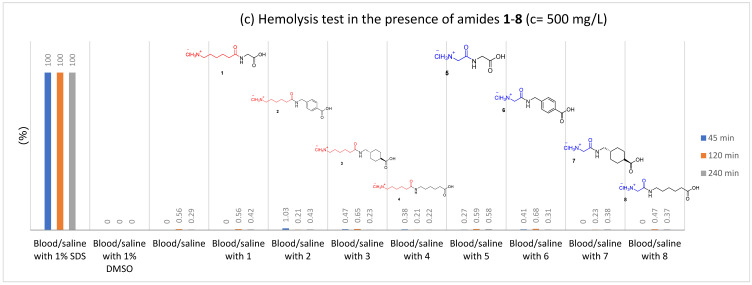
The influence of amides **1**–**8** on the stability of erythrocytes (hemolysis test); (**a**) c = 5 mg/L; (**b**) c = 50 mg/L; (**c**) c = 500 mg/L.

To study the interactions of the synthesized amides **1**–**8** with blood cells, we examined their impact on erythrocytes. None of the synthesized compounds **1**–**8** caused hemolysis at any concentrations (5, 50, or 500 mg/L), even after 4 h. Hemolysis did not exceed 1% with any of the compounds. Even at a concentration of 500 mg/L, hemolysis was less than 1% in the initial incubation period up to 4 h. Figure 7a–c compare results from the hemolysis tests for all synthetized amides **1**–**8** at concentrations 5, 50, and 500 mg/L. As can be seen, there is no difference in the hemolysis parameter between blood cells samples exposed to the amides. Other graphs showing results of the hemolysis tests for all single amides **1**–**8** are given in the Supporting Information (Figures S33–S40).

#### 2.2.2. Clotting Assays and Determination of Fibrinolytic Efficiency

The hemostatic properties of the synthesized amides were evaluated based on a comprehensive study on their effects on both the human blood plasma coagulation process and its physiological counterpart, i.e., fibrinolysis. The efficiency of the examined amides was tested using blood clotting times, well-known diagnostic parameters, and the clot formation and fibrinolysis (CFF) assay, which provides data not only on the plasma coagulation process but also on the rate of fibrinolysis. The hemostatic activity of the examined amides was established in comparison with an antifibrinolytic drug, tranexamic acid. In our tests, this compound slightly shortened the prothrombin time (PT) and the activated partial thromboplastin time (aPTT), with no effects on the thrombin time TT. All synthesized amides **1**–**8** were evaluated in clotting tests, including the prothrombin time (PT), the activated partial thromboplastin time (aPTT), and the (TT). The compounds were tested at three concentrations: 10, 25, and 50 mg/L. TA was used as a reference compound, and untreated blood plasma was used as a control sample. The results for all clotting times are presented as a percentage of the control.

##### Prothrombin Time (PT)

**Figure 8:**
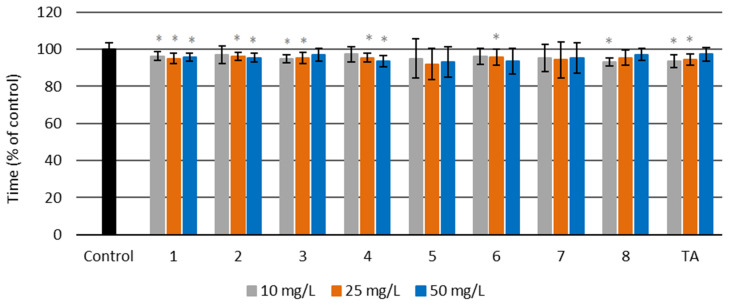
Prothrombin time (PT) for amides **1**–**8** and tranexamic acid (TA) at three different concentrations—10, 25, and 50 mg/L—and for the control sample of human blood plasma (untreated with the examined amides) (* *p* < 0.05).

The prothrombin times for untreated plasma and plasma treated with amides ranged from 14.1 to 15.2 s (see Supporting Information, Figure S41). The results are presented as a percentage of the control, which was plasma without amides and TA (Figure 8). Figure 8 shows that prothrombin time was slightly but statistically significantly reduced in plasma with amides **1**–**4** and **8,** as well as with tranexamic acid. Amide **1** at all concentrations resulted in a significantly decreased prothrombin time, as did amides **2** and **4** at two higher concentrations (25 and 50 mg/L). Amide **3** and TA at two lower concentrations (10 and 25 mg/L) decreased the prothrombin time, which fell only slightly with amide **8** at the lowest concentration (10 mg/L). The prothrombin time was most effectively lowered by amide **8** at a concentration of 10 mg/L.

##### Activated Partial Thromboplastin Time (aPTT)

**Figure 9:**
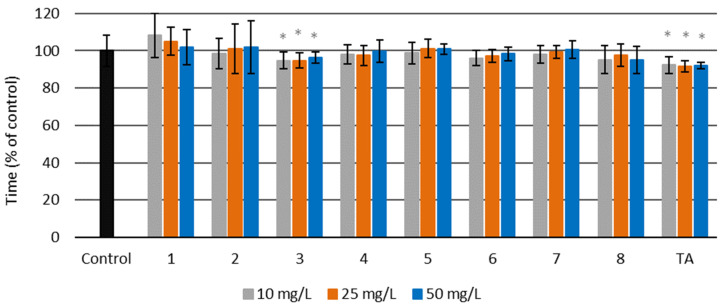
Activated partial thromboplastin time (aPTT) for amides **1**–**8** and tranexamic acid (TA) at three different concentrations—10, 25, and 50 mg/L—and for the control sample of human blood plasma (untreated with the examined amides) (* *p* < 0.05).

For synthesized amides **1**–**8**, the activated partial thromboplastin time (aPTT) was in the range of 51.2–58.0 s (see Supporting Information, Figure S42). These results are presented as a percentage of the control in the Figure 9. Only two of the tested substances (amid **3** and TA) significantly decreased the aPTT time. The other compounds did not cause statistically significant changes in aPTT times. Amide **1** appears to increase aPTT time, but the changes are not statistically significant.

##### Thrombin Time (TT)

**Figure 10:**
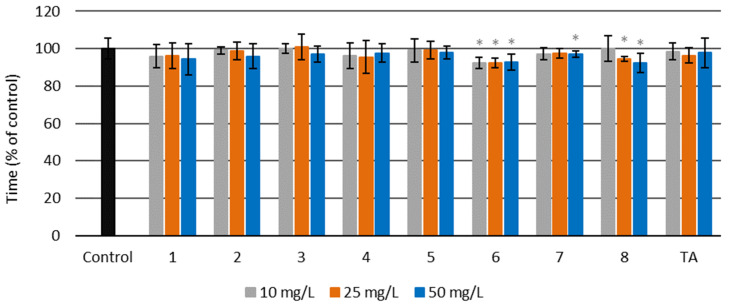
Thrombin time (TT) for amides **1**–**8** and tranexamic acid (TA) at three different concentrations—10, 25 and 50 mg/L—and for the control sample of human blood plasma (untreated with the examined amides) (* *p* < 0.05).

The thrombin time (TT) for all synthesized amides **1**–**8** was between 14.6 and 16.9 s, and for the control sample TT, it was 15.7 s (see Supporting Information, Figure S43). The results are presented as a percentage of the control in Figure 10. Only amide **6** at all used concentrations and amide **8** (c = 25, and 50 mg/L) had shorter TT than the control sample. There were no significant changes in TT for the other tested amides.

##### Clot Formation and Fibrinolysis (CFF) Assay 

**Figure 11:**
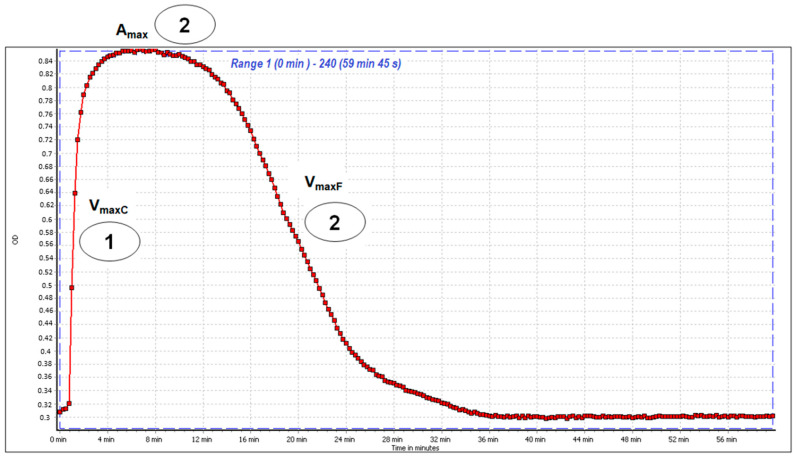
An exemplary curve of blood plasma coagulation and clot lysis recorded during the clot formation and fibrinolysis (CFF) assay. The first step in the CFF test involves activation of the blood plasma coagulation cascade and fibrin clot formation (1). It is characterized by the maximal velocity of the fibrin polymerization parameter (V_maxC_). The maximal absorbance (A_max_) peak corresponds to the fibrin stabilization phase and is an indicator of fibrin clot thickness (2). The third step of the CFF assay covers the activation of fibrinolytic mechanisms and fibrin clot degradation (3) and is described by the maximal velocity of clot lysis (V_maxF_).

The CFF assay enables analysis of both the blood plasma coagulation process and fibrinolysis efficiency. The thrombin enzyme in the reagent mixture initiates blood plasma fibrinogen polymerization, leading to an increase in absorbance in the sample until the maximal absorbance (A_max_) is attained. The fibrinolytic activity of blood plasma is induced by the tissue-type plasminogen activator (t-PA). The activation of fibrinolysis in the tested samples leads to fibrin clot degradation and decreases the absorbance to the baseline. An exemplary plot obtained in the assay is presented in Figure 11.

In the CFF assay, some of the examined compounds showed pro-coagulant and antifibrinolytic activity (Table 1). Mild pro-coagulant effects (measured as V_maxC_) were observed mainly for **5**, **1**, and **2**, while **8** and TA (a reference antifibrinolytic drug) did not influence the blood plasma polymerization process. Most of the examined substances showed significant changes in the A_max_ parameter, indicating that their presence may enhance fibrin clot thickness. For **5** and **7**, the A_max_ parameter was significantly higher across the full range of tested concentrations (10–50 µg/mL). Fibrinolytic activity was diminished by most of the examined compounds at a concentration of 50 µg/mL, except for **1**, which had no statistically significant effect (*p* > 0.05). The most effective reducers of blood plasma fibrinolytic activity were **4** and **7**, which decreased V_maxF_ by over 30–40%.

#### 2.2.3. Cytotoxicity and Genotoxicity Analysis

**Figure 12:**
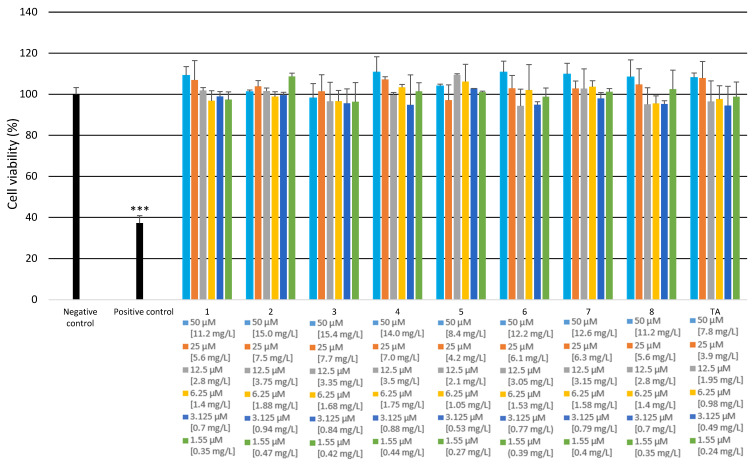
Cytotoxicity of the examined amides on SC cells. The XTT assay was used to assess the cytotoxicity of the tested amides. All of the experiments were performed in triplicate. Data are expressed as mean ± SD (*n* = 3), *** *p* < 0.001 versus the negative control.

**Figure 13:**
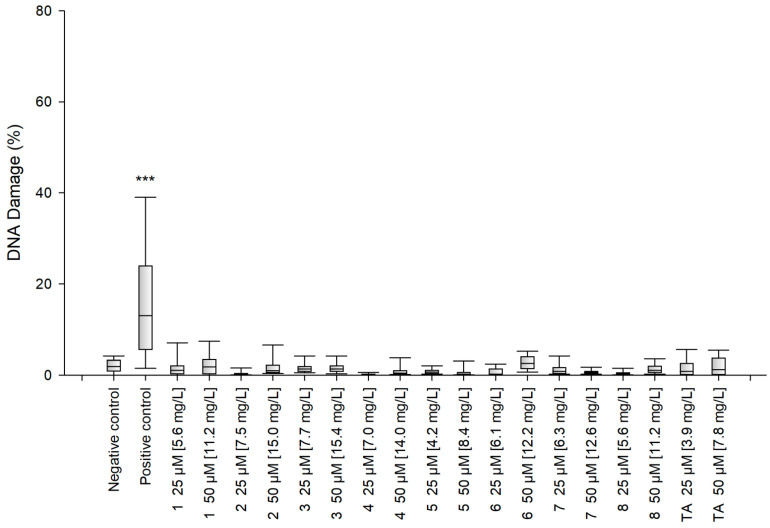
Genotoxicity of the tested compounds. Statistical analysis was based on the results of three independent tests. The differences were statistically significant as follows: *** *p* < 0.001 versus the negative control.

The cytotoxicity and genotoxicity of all the synthesized amides **1**–**8** were evaluated, in the range from 50 to 1.5 μM, on a commercially available monocyte/macrophage peripheral blood cell line (SC). Untreated cells cultured in a complete medium were used as a negative control. SC cells treated with 100% DMSO were used as a positive control. The results are presented in Figure 12 and Figure 13. As can be seen in Figure 12, none of the investigated amides **1**–**8** had any significant cytotoxic effect on SC cells at any concentration. The results for the cells incubated with the tested compounds are very similar to those obtained for the negative control. The cytotoxicity of TA was similar to the cytotoxicity of the synthesized amides **1**–**8**.

The alkaline version of the comet assay was used to assess the level of DNA damage caused by the tested compounds. The results show that after 48 h incubation, none of the synthesized amides **1**–**8** had caused significant DNA damage in the SC cells at any of the tested concentrations (Figure 13). The genotoxicity of TA was similar to the genotoxicity of amides **1**–**8**.

## 3. Materials and Methods

NMR spectra were measured on a Bruker Avance II Plus (Bruker Corporation, Billerica, MA, USA) spectrometer (700 MHz for ^1^H-NMR and 176 MHz for ^13^C-NMR) in CDCl_3_ solution. ^1^H and ^13^C-NMR spectra were referenced according to the residual peak of the solvent based on literature data. Chemical shifts (*d*) were reported in ppm and coupling constants (*J*) were reported in Hz. ^13^C-NMR spectra were proton-decoupled. Flash chromatography was performed using a glass column packed with Baker silica gel (30–60 μm). For TLC, silica gel was used with a 254 nm indicator on Al foils (Sigma-Aldrich, St. Louis, MO, USA). All reagents and solvents were purchased and used as obtained from Sigma-Aldrich (Poznan, Poland). Melting points were obtained using a Büchi SMP-20 apparatus. Mass spectrometry analysis was performed on a Bruker microOTOF-QIII (Bruker Corporation, Billerica, MA, USA) equipped with electrospray ionization mode and a time-of-flight detector (TOF). IR spectra were measured on an FT-IR Alpha Bruker (ATR) instrument in cm^−1^.

### 3.1. General Procedures for the Synthesis of N-Boc Amides ***18***–***25***

6-(Boc-amino)caproic acid (**9**) (0.231 g, 1 mmol, 1 equiv.) or *N*-Boc glycine (**10**) (0.175 g, 1 mmol, 1 equiv.) were dissolved in dichloromethane (5 mL) in a 25 mL round-bottom flask. The solution was mixed and cooled in an ice-water bath. Then, DMT/NMM/TsO^−^ (**11**) (0.414 g, 1 mmol, 1 equiv.) and NMM (0.11 mL, 1 mmol, 1 equiv.) were added. In the case of compound **9**, the reaction was controlled by TLC (DCM/acetone 10:1). For compound **10**, the reaction was mixed for 30 min. After that, the appropriate hydrochloride (glycine *tert*-butyl ester hydrochloride (**14**) (0.167 g, 1 mmol, 1 equiv.), ethyl ester of 4-(aminomethyl)benzoic acid hydrochloride (**15**) (0.215 g, 1 mmol, 1 equiv.), ethyl ester of *trans*-4-(aminomethyl)cyclohexanecarboxylic acid hydrochloride (**16**) (0.222 g, 1 mmol, 1 equiv.), or ethyl ester of 6-aminocaproic acid hydrochloride (**17**) (0.195 g, 1 mmol, 1 equiv.) was added with NMM (0.12 mL, 1.1 mM) to the solution, and the reaction was left to continue overnight at room temperature. 

Each solution was diluted with DCM (50 mL) and washed with H_2_O (5 mL), 1 M NaHSO_4_ (2 × 5 mL), H_2_O (5 mL), 1 M NaHCO_3_ (2 × 5 mL), and H_2_O (5 mL). The organic layer was dried under anhydrous MgSO_4_; then, the solvent was evaporated under reduced pressure. Amides **18**–**25** were isolated after purification by flash chromatography on silica gel or by crystallization.

*Tert-butyl 2-(6-((tert-butoxycarbonyl)amino)hexanamido)acetate* (**18**)

White solid, mp 64–65 °C (lit. 59–61 °C). Yield 71% (0.244 g) after crystallization with hexane. ^1^H-NMR (CDCl_3_, 700 MHz): *δ* = 5.93 (bs, 1 H, N*H*), 4.56 (bs, 1 H, N*H*), 3.92 (d, *J*_HH_ = 5.0 Hz, 2 H, C*H*_2_), 3.11 (bs, 2 H, C*H*_2_), 2.23 (t, *J*_HH_ = 7.5 Hz, 2 H, C*H*_2_), 1.68–1.65 (m, 2 H, C*H*_2_), 1.51–1.48 (m, 2 H, C*H*_2_), 1.47 (s, 9 H, (C*H*_3_)_3_C), 1.43 (s, 9 H, (C*H*_3_)_3_C), 1.37–1.33 (m, 2 H, C*H*_2_). ^13^C-NMR (CDCl_3_, 176 MHz): *δ* = 172.9 (s, *C*O), 169.4 (s, *C*O), 156.1 (s, *C*O), 82.3 (s, *C*(CH_3_)_3_), 79.1 (s, *C*(CH_3_)_3_), 42.1 (s, *C*H_2_), 40.3 (s, *C*H_2_), 36.3 (s, *C*H_2_), 29.9 (s, *C*H_2_), 28.5 (s, 3 × *C*H_3_), 28.1 (s, 3 × *C*H_3_), 26.4 (s, *C*H_2_), 25.2 (s, *C*H_2_). IR (ATR): 3340, 2937, 2869, 1742 (CO), 1689 (CONH), 1661 (CO), 1543, 1522, 1365, 1244, 1160 (COO), 855, 620, 575 cm^−1^. HRMS: 344.2309 ([M]^+^, C_17_H_32_N_2_O_5_^+^; calc. 344.2311). The analytical data are in agreement with those reported previously in the literature [49].

*Ethyl 4-((6-((tert-butoxycarbonyl)amino)hexanamido)methyl)benzoate* (**19**)

White solid, mp 107–108 °C. Yield 66% (0.260 g) after crystallization hexane/ethyl acetate. ^1^H-NMR (CDCl_3_, 700 MHz): *δ* = 7.99 (d, *J*_HH_ = 8.4 Hz, 2 H, 2 × C*H*_Ar_), 7.32 (d, *J*_HH_ = 8.5 Hz, 2 H, 2 × C*H*_Ar_), 5.93 (bs, 1 H, N*H*), 4.55 (bs, 1 H, N*H*), 4.49 (d, *J*_HH_ = 5.9 Hz, 2 H, C*H*_2_), 4.36 (q, *J*_HH_ = 7.1 Hz, 2 H, OC*H*_2_), 3.12–3.07 (m, 2 H, C*H*_2_), 2.23 (t, *J*_HH_ = 7.5 Hz, 2 H, C*H*_2_), 1.68 (qw, *J*_HH_ = 7.6 Hz, 2 H, C*H*_2_), 1.49 (qw, *J*_HH_ = 7.1 Hz, 2 H, C*H*_2_), 1.42 (s, 9 H, (C*H*_3_)_3_C), 1.38 (t, *J*_HH_ = 7.1 Hz, 3 H, OCH_2_C*H*_3_), 1.37–1.33 (m, 2 H, C*H*_2_). ^13^C-NMR (CDCl_3_, 176 MHz): *δ* = 172.9 (s, *C*O), 166.4 (s, *C*O), 156.1 (s, *C*O), 143.7 (s, *C*_Ar_C), 130.0 (s, 2 × *C*_Ar_H), 129.7 (s, *C*_Ar_), 127.6 (s, 2 × *C*_Ar_H), 79.2 (s, *C*(CH_3_)_3_), 61.1 (s, O*C*H_2_CH_3_), 43.3 (s, *C*H_2_), 40.3 (s, *C*H_2_), 36.5 (s, *C*H_2_), 29.8 (s, *C*H_2_), 28.4 (s, 3 × *C*H_3_), 26.5 (s, *C*H_2_), 25.3 (s, *C*H_2_), 14.4 (OCH_2_*C*H_3_). IR (ATR): 3348, 3331, 2933, 2860, 1724 (CO), 1684 (CONH), 1639 (CO), 1532, 1248, 1173 (COO), 1133, 1040, 992, 643, 614 cm^−1^. HRMS: 392.2315 ([M]^+^, C_21_H_32_N_2_O_5_^+^; calc. 392.2311). New compound.

*(1s,4s)-Ethyl 4-((6-((tert-butoxycarbonyl)amino)hexanamido)methyl)-cyclohexanecarboxylate* (**20**)

White solid, mp 66–67 °C. Yield 71% (0.279 g) after flash chromatography ethyl acetate/hexane 2:1. ^1^H-NMR (CDCl_3_, 700 MHz): *δ* = 5.58 (bs, 1 H, N*H*), 4.56 (bs, 1 H, N*H*), 4.08 (q, *J*_HH_ = 7.1 Hz, 2 H, OC*H*_2_CH_3_), 3.10 (t, *J*_HH_ = 6.4 Hz, 4 H, 2 × C*H*_2_), 2.20 (tt, *J*_HH_ = 12.3 Hz, *J*_HH_ = 3.6 Hz, 1 H, C*H*), 2.17–2.15 (m, 2 H, C*H*_2_), 2.00–1.98 (m, 2 H, C*H*_2_), 1.81–1.79 (m, 2 H, C*H*_2_), 1.64 (qw, *J*_HH_ = 7.6 Hz, 2 H, C*H*_2_), 1.50–1.46 (m, 2 H, C*H*_2_), 1.45–1.44 (m, 1 H, C*H*), 1.42 (s, 9 H, (C*H*_3_)_3_C), 1.41–1.37 (m, 2 H, C*H*_2_), 1.35–1.31 (m, 2 H, C*H*_2_), 1.23 (t, *J*_HH_ = 7.1 Hz, 3 H, OCH_2_C*H*_3_), 0.96 (qd, *J*_HH_ = 13.2 Hz, *J*_HH_ = 3.5 Hz, 2 H, C*H*_2_). ^13^C-NMR (CDCl_3_, 176 MHz): *δ* = 175.9 (s, *C*O), 173.0 (s, *C*O), 156.1 (s, *C*O), 79.1 (s, *C*(CH_3_)_3_), 60.2 (s, O*C*H_2_CH_3_), 45.3 (s, *C*H_2_), 43.3 (s, *C*H), 40.3 (s, *C*H_2_), 37.5 (s, *C*H), 36.7 (s, *C*H_2_), 29.9 (s, 2 × *C*H_2_), 29.8 (s, *C*H_2_), 28.5 (s, 2 × *C*H_2_), 28.4 (s, 3 × *C*H_3_), 26.5 (s, *C*H_2_), 25.4 (s, *C*H_2_), 14.3 (OCH_2_*C*H_3_). IR (ATR): 3349, 3331, 2933, 2860, 1724 (CO), 1684 (CONH), 1640 (CO), 1532, 1249, 1174 (COO), 1134, 1040, 643, 615 cm^−1^. HRMS: 399.2851 ([M + H]^+^, C_21_H_39_N_2_O_5_^+^; calc. 399.2853). New compound.

*Ethyl 6-(6-((tert-butoxycarbonyl)amino)hexanamido)hexanoate* (**21**)

Colorless oil. Yield 77% (0.293 g) after flash chromatography hexane/ethyl acetate 1:1. ^1^H-NMR (CDCl_3_, 700 MHz): *δ* = 5.67 (bs, 1 H, N*H*), 4.61 (bs, 1 H, N*H*), 4.10 (q, *J*_HH_ = 7.1 Hz, 2 H, OC*H*_2_CH_3_), 3.23–3.20 (m, 2 H, C*H*_2_), 3.10–3.05 (m, 2 H, C*H*_2_), 2.27 (t, *J*_HH_ = 7.4 Hz, 2 H, C*H*_2_), 2.13 (t, *J*_HH_ = 7.5 Hz, 2 H, C*H*_2_), 1.64–1.59 (m, 4 H, 2 × C*H*_2_), 1.51–1.44 (m, 4 H, 2 × C*H*_2_), 1.41 (s, 9 H, (C*H*_3_)_3_C), 1.35–1.29 (m, 4 H, 2 × C*H*_2_), 1.23 (t, *J*_HH_ = 7.1 Hz, 3 H, OCH_2_C*H*_3_). ^13^C-NMR (CDCl_3_, 176 MHz): *δ* = 173.7 (s, *C*O), 172.9 (s, *C*O), 156.1 (s, *C*O), 79.1 (s, *C*(CH_3_)_3_), 60.3 (s, O*C*H_2_CH_3_), 40.4 (s, *C*H_2_), 39.2 (s, *C*H_2_), 36.7 (s, *C*H_2_), 34.2 (s, *C*H_2_), 29.8 (s, *C*H_2_), 29.3 (s, *C*H_2_), 28.5 (s, 3 × *C*H_3_), 26.5 (s, *C*H_2_), 26.4 (s, *C*H_2_), 25.4 (s, *C*H_2_), 24.5 (s, *C*H_2_), 14.3 (OCH_2_*C*H_3_). IR (ATR): 3383, 2933, 1728 (CO), 1701 (CONH), 1637 (CO), 1541, 1512, 1239, 1169 (COO), 1034, 584 cm^−1^. HRMS: 373.2690 ([M + H]^+^, C_19_H_37_N_2_O_5_^+^; calc. 373.2697). New compound.

*Tert-butyl 2-(2-((tert-butoxycarbonyl)amino)acetamido)acetate[N-Boc-Gly-Gly-OtBu]* (**22**)

Colorless oil. Yield 68% (0.195 g) after flash chromatography hexane/ethyl acetate 1:1. ^1^H-NMR (CDCl_3_, 700 MHz): *δ* = 6.67 (bs, 1 H, N*H*), 5.30 (bs, 1 H, N*H*), 3.93 (d, *J*_HH_ = 5.1 Hz, 2 H, C*H*_2_), 3.83 (d, *J*_HH_ = 4.5 Hz, 2 H, C*H*_2_), 1.45 (s, 9 H, (C*H*_3_)_3_C), 1.43 (s, 9 H, (C*H*_3_)_3_C). ^13^C-NMR (CDCl_3_, 176 MHz): *δ* = 169.7 (s, *C*O), 168.9 (s, *C*O), 156.1 (s, *C*O), 82.4 (s, *C*(CH_3_)_3_), 80.3 (s, *C*(CH_3_)_3_), 44.2 (s, *C*H_2_), 42.1 (s, *C*H_2_), 28.4 (s, 3 × *C*H_3_), 28.1 (s, 3 × *C*H_3_). IR (ATR): 3355, 3272, 2977, 2935, 1733 (CO), 1673 (CONH), 1506, 1366, 1224, 1151 (COO), 1049, 1033, 945,847 cm^−1^. HRMS: 289.1755 ([M + H]^+^, C_13_H_25_N_2_O_5_^+^; calc. 289.1758).The analytical data are in agreement with those reported previously in the literature [50].

*Ethyl 4-((2-((tert-butoxycarbonyl)amino)acetamido)methyl)benzoate* (**23**)

White solid, mp 90 °C. Yield 69% (0.232 g) after crystallization hexane/ethyl acetate. ^1^H-NMR (CDCl_3_, 700 MHz): *δ* = 7.92 (d, *J*_HH_ = 8.3 Hz, 2 H, 2 × C*H*_Ar_), 7.27 (d, *J*_HH_ = 8.3 Hz, 2 H, 2 × C*H*_Ar_), 7.05 (bs, 1 H, N*H*), 5.48 (t, J_HH_ = 5.2 Hz, 1 H, N*H*), 4.44 (d, *J*_HH_ = 6.1 Hz, 2 H, C*H*_2_), 4.32 (q, *J*_HH_ = 7.1 Hz, 2 H, OC*H*_2_CH_3_), 3.81 (bs, 2 H, C*H*_2_), 1.38 (s, 9 H, (C*H*_3_)_3_C), 1.35 (t, *J*_HH_ = 7.1 Hz, 3 H, OCH_2_C*H*_3_). ^13^C-NMR (CDCl_3_, 176 MHz): *δ* = 169.8 (s, *C*O), 166.4 (s, *C*O), 156.3 (s, *C*O), 143.2 (s, *C*_Ar_), 129.9 (s, 2 × *C*_Ar_H), 129.6 (s, *C*_Ar_), 127.3 (s, 2 × *C*_Ar_H), 80.3 (s, *C*(CH_3_)_3_), 61.0 (s, O*C*H_2_CH_3_), 44.5 (s, *C*H_2_), 43.1 (s, *C*H_2_), 28.3 (s, 3 × *C*H_3_), 14.3 (OCH_2_*C*H_3_). IR (ATR): 3311, 2980, 2931, 1707 (CO), 1654 (CONH), 1611 (CO), 1531, 1390, 1364, 1267, 1170 (COO), 1101, 1020, 861, 749, 697, 661, 581 cm^−1^. HRMS: 337.1756 ([M + H]^+^, C_17_H_25_N_2_O_5_^+^; calc. 337.1758). New compound.

*(1s,4s)-Ethyl 4-((2-((tert-butoxycarbonyl)amino)acetamido)methyl)cyclohexanecarboxylate* (**24**)

Colorless oil. Yield 62% (0.213 g) after flash chromatography hexane/ethyl acetate 1:1. ^1^H-NMR (CDCl_3_, 700 MHz): *δ* = 6.44 (bs, 1 H, N*H*), 5.32 (bs, 1 H, N*H*), 4.08 (q, *J*_HH_ = 7.1 Hz, 2 H, OC*H*_2_CH_3_), 3.74 (d, *J*_HH_ = 5.7 Hz, 2 H, C*H*_2_), 3.10 (t, *J*_HH_ = 6.5 Hz, 2 H, C*H*_2_), 2.18 (tt, *J*_HH_ = 12.2 Hz, *J*_HH_ = 3.6 Hz, 1 H, C*H*), 1.98–1.95 (m, 2 H, C*H*_2_), 1.80–1.78 (m, 2 H, C*H*_2_), 1.47–1.44 (m, 1 H, C*H*), 1.43 (s, 9 H, (C*H*_3_)_3_C), 1.37 (qd, *J*_HH_ = 13.1 Hz, *J*_HH_ = 3.4 Hz, 2 H, C*H*_2_), 1.22 (t, *J*_HH_ = 7.1 Hz, 3 H, OCH_2_C*H*_3_), 0.95 (qd, *J*_HH_ = 13.3 Hz, *J*_HH_ = 3.5 Hz, 2 H, C*H*_2_). ^13^C-NMR (CDCl_3_, 176 MHz): *δ* = 175.9 (s, *C*O), 169.6 (s, *C*O), 156.3 (s, *C*O), 80.3 (s, *C*(CH_3_)_3_), 60.2 (s, O*C*H_2_CH_3_), 45.3 (s, *C*H_2_), 44.6 (s, *C*H_2_), 43.2 (s, *C*H), 37.4 (s, *C*H), 29.8 (s, 2 × *C*H_2_), 28.4 (s, 2 × *C*H_2_), 28.3 (s, 3 × *C*H_3_), 14.3 (OCH_2_*C*H_3_). IR (ATR): 3311, 2979, 2931, 1708 (CO), 1654 (CONH), 1611 (CO), 1450, 1390, 1363, 1266, 1170 (COO), 1100, 1020, 748, 697, 664, 592 cm^−1^. HRMS: 343.2223 ([M + H]^+^, C_17_H_31_N_2_O_5_^+^; calc. 343.2227). New compound.

*Ethyl 6-(2-((tert-butoxycarbonyl)amino)acetamido)hexanoate* (**25**) 

Colorless oil. Yield 66% (0.207 g) after flash chromatography hexane/ethyl acetate 1:1. ^1^H-NMR (CDCl_3_, 700 MHz): *δ* = 6.29 (bs, 1 H, N*H*), 5.25 (bs, 1 H, N*H*), 4.10 (q, *J*_HH_ = 7.1 Hz, 2 H, OC*H*_2_CH_3_), 3.75 (d, *J*_HH_ = 5.6 Hz, 2 H, C*H*_2_), 3.25 (q, *J*_HH_ = 6.8 Hz, 2 H, C*H*_2_), 2.27 (t, *J*_HH_ = 7.4 Hz, 2 H, C*H*_2_), 1.62 (qw, *J*_HH_ = 7.5 Hz, 2 H, C*H*_2_), 1.51 (qw, *J*_HH_ = 7.4 Hz, 2 H, C*H*_2_), 1.43 (s, 9 H, (C*H*_3_)_3_C), 1.33 (qw, *J*_HH_ = 7.7 Hz, 2 H, C*H*_2_), 1.23 (t, *J*_HH_ = 7.1 Hz, 3 H, OCH_2_C*H*_3_). ^13^C-NMR (CDCl_3_, 176 MHz): *δ* = 173.7 (s, *C*O), 169.4 (s, *C*O), 156.2 (s, *C*O), 80.3 (s, *C*(CH_3_)_3_), 60.4 (s, O*C*H_2_CH_3_), 44.6 (s, *C*H_2_), 39.2 (s, *C*H_2_), 34.2 (s, *C*H_2_), 29.2 (s, *C*H_2_), 28.4 (s, 3 × *C*H_3_), 26.4 (s, *C*H_2_), 24.5 (s, *C*H_2_), 14.3 (OCH_2_*C*H_3_). IR (ATR): 3305, 2976, 2933 (CO), 1714 (CONH), 1656 (CO), 1522, 1454, 1367, 1245, 1161 (COO), 1098, 1029, 944, 863 cm^−1^. HRMS: 317.2070 ([M + H]^+^, C_15_H_29_N_2_O_5_^+^; calc. 317.2071). New compound.

### 3.2. General Procedure for Synthesis Hydrochlorides of Amides ***1*** and ***5***

The solution of amide **18** or **22** (1 mmol, 1 equiv.) in dioxane (2 mL) was stirred and cooled in an ice bath in a 10 mL round-bottom flask equipped with a magnetic bar. Then, 4 M HCl/dioxane (8 mL) was added, and the reaction was carried out overnight at rt. The next day, the solvent was evaporated, and the residue was co-evaporated with dioxane (3 × 5 mL). Final products **1** and **5** were isolated in semi-solid form after precipitating by washing with cold Et_2_O (10 mL) and filtration.

*2-(6-Aminohexanamido)acetic acid hydrochloride* (**1**)

Yield 93% (0.208 g). ^1^H-NMR (D_2_O, 700 MHz): *δ* = 4.00 (s, 2 H, C*H*_2_), 3.01 (t, *J*_HH_ = 7.6 Hz, 2 H, C*H*_2_), 2.36 (t, *J*_HH_ = 7.4 Hz, 2 H, C*H*_2_), 1.71–1.64 (m, 4 H, 2 × C*H*_2_), 1.42 (qw, *J*_HH_ = 7.7 Hz, 2 H, C*H*_2_). ^13^C-NMR (D_2_O, 176 MHz): *δ* = 177.3 (s, *C*O), 173.4 (s, *C*O), 41.1 (s, *C*H_2_), 39.3 (s, *C*H_2_), 35.0 (s, *C*H_2_), 26.3 (s, *C*H_2_), 25.0 (s, *C*H_2_), 24.5 (s, *C*H_2_). IR (ATR): 3273, 3026 (OH), 2936 (OH), 1736 (CO), 1642 (CONH), 1559, 1512, 1395, 1165, 1119, 1034, 869, 763, 715 cm^−1^. HRMS: 225.1002 ([M + H]^+^, C_8_H_18_ClN_2_O_3_^+^; calc. 225.1000). New compound.

*Glycylglycine hydrochloride* (**5**)

White solid, mp 123–126 °C. Yield 97% (0.158 g). ^1^H-NMR (D_2_O, 700 MHz): *δ* =4.10 (s, 2 H, C*H*_2_), 3.93 (s, 2 H, C*H*_2_). ^13^C-NMR (D_2_O, 176 MHz): *δ* = 173.3 (s, *C*O), 167.6 (s, *C*O), 41.1 (s, *C*H_2_), 40.5 (s, *C*H_2_). **IR** (ATR): 3270, 3052 (OH), 2942 (OH), 1740 (CO), 1671 (CONH), 1580, 1483, 1212, 1094, 901, 869, 657, 585, 559, 536 cm^−1^. **HRMS**: 169.0372 ([M + H]^+^, C_4_H_10_ClN_2_O_3_^+^; calc. 169.0374). A known compound, not described in the literature.

### 3.3. General Procedure for Synthesis Hydrochlorides of Amides ***2***–***4*** and ***5***–***7***

In a 25 mL round-bottom flask, amides **19**–**21** or **23**–**25** (1 mmol, 1 equiv.) were dissolved in methanol (8 mL), and 1 M NaOH (2 mL) was added. The reaction was carried out in reflux for 2 h. Next, the reaction was diluted in water (10 mL) and washed with Et_2_O (2 × 20 mL). The water phase was acidified to pH 2–3 using 1 M KHSO_4_ and extracted with EtOAc (3 × 20 mL). The combined organic layers were dried under anhydrous MgSO_4_ and concentrated in a vacuum, giving intermediate products **26**–**28** and **29**–**31**. Subsequently, compounds **26**–**28** and **29**–**31** were dissolved in dioxane (2 mL), stirred, and cooled in an ice bath, following which 4M HCl/dioxane (8 mL) was added. The ice bath was removed, and stirring was continued for 1 h at rt. Then, the solvent was evaporated, and the residue was co-evaporated with dioxane (3 × 5 mL). The final products **2**–**4** and **5**–**7** were isolated as a white solid after precipitation by washing with cold Et_2_O (10 mL) and filtration.

*4-((6-Aminohexanamido)methyl)benzoic acid hydrochloride* (**2**)

White solid, mp 199–201 °C. Yield 99% (0.297 g). ^1^H-NMR (D_2_O, 700 MHz): *δ* = 7.99 (d, *J*_HH_ = 8.3 Hz, 2 H, 2 × C*H*_Ar_), 7.42 (d, *J*_HH_ = 8.2 Hz, 2 H, 2 × C*H*_Ar_), 4.45 (s, 2 H, C*H*_2_), 3.00 (t, *J*_HH_ = 7.7 Hz 2 H, C*H*_2_), 2.36 (t, *J*_HH_ = 7.4 Hz, 2 H, C*H*_2_), 1.71–1.65 (m, 4 H, 2 × C*H*_2_), 1.39 (qw, *J*_HH_ = 7.7 Hz, 2 H, C*H*_2_). ^13^C-NMR (D_2_O, 176 MHz): *δ* = 176.7 (s, *C*O), 170.3 (s, *C*O), 144.1 (s, *C*_Ar_), 130.0 (s, 2 × *C*_Ar_H), 128.5 (s, *C*_Ar_), 127.2 (s, 2 × *C*_Ar_H), 42.7 (s, *C*H_2_), 39.3 (s, *C*H_2_), 35.4 (s, *C*H_2_), 26.4 (s, *C*H_2_), 25.1 (s, *C*H_2_), 24.8 (s, *C*H_2_). IR (ATR): 3294, 2934 (OH), 2055 (Ar), 1686 (CO), 1635 (CONH), 1609, 1524, 1425, 1322, 1292, 1206, 937, 695, 543 cm^−1^. HRMS: 301.1319 ([M + H]^+^, C_14_H_22_ClN_2_O_3_^+^; calc. 301.1313). New compound.

*6-((((1s,4s)-4-Carboxycyclohexyl)methyl)amino)-6-oxohexan-1-aminium chloride* (**3**)

White solid, mp 195–197 °C. Yield 94% (0.287 g). ^1^H-NMR (D_2_O, 700 MHz): 3.08 (d, *J*_HH_ = 6.8 Hz, 2 H, C*H_2_*), 3.02 (t, *J*_HH_ = 7.6 Hz, 2 H, C*H*_2_), 2.36 (tt, *J*_HH_ = 12.2 Hz, *J*_HH_ = 3.5 Hz, 1 H, C*H*), 2.30 (t, *J*_HH_ = 7.4 Hz, 2 H, C*H*_2_), 2.03–2.01 (m, 2 H, C*H*_2_), 1.84–1.82 (m, 2 H, C*H*_2_), 1.71 (qw, *J*_HH_ = 7.7 Hz, 2 H, C*H*_2_), 1.66 (qw, *J*_HH_ = 7.5 Hz, 2 H, C*H*_2_), 1.55–1.49 (m, 1 H, C*H*), 1.44–1.38 (m, 4 H, 2 × C*H*_2_), 1.03 (qd, *J*_HH_ = 13.1 Hz, *J*_HH_ = 3.4 Hz, 2 H, C*H*_2_). ^13^C-NMR (D_2_O, 176 MHz): *δ* = 181.2 (s, *C*O), 176.6 (s, *C*O), 45.2 (s, *C*H_2_), 42.9 (s, *C*H), 39.3 (s, *C*H_2_), 36.6 (s, *C*H), 35.5 (s, *C*H_2_), 29.1 (s, 2 × *C*H_2_), 28.1 (s, 2 × *C*H_2_), 26.4 (s, *C*H_2_), 25.1 (s, *C*H_2_), 24.9 (s, *C*H_2_). IR (ATR): 3299, 2928 (OH), 2853 (OH), 1712 (CO), 1636 (CONH), 1560, 1251, 1222, 1178, 1131, 670 cm^−1^. HRMS: 307.1785 ([M + H]^+^, C_14_H_28_ClN_2_O_3_^+^; calc. 307.1783). New compound.

*6-(6-Aminohexanamido)hexanoic acid hydrochloride* (**4**)

White solid, mp 82–83 °C (lit. 78–80 °C [51]). Yield 93% (0.260 g). ^1^H-NMR (D_2_O, 700 MHz): *δ=* 3.19 (t, *J*_HH_ = 6.8 Hz, 2 H, C*H*_2_), 3.00 (t, *J*_HH_ = 7.6 Hz, 2 H, C*H*_2_), 2.40 (t, *J*_HH_ = 7.3 Hz, 2 H, C*H*_2_), 2.26 (t, *J*_HH_ = 7.3 Hz, 2 H, C*H*_2_), 1.69 (qw, *J*_HH_ = 7.7 Hz, 2 H, C*H*_2_), 1.63 (qw, *J*_HH_ = 7.7 Hz, 4 H, 2 × C*H*_2_), 1.53 (qw, *J*_HH_ = 7.0 Hz, 2 H, C*H*_2_), 1.41–1.33 (m, 4 H, 2 × C*H*_2_). ^13^C-NMR (D_2_O, 176 MHz): *δ* = 178.9 (s, *C*O), 176.5 (s, *C*O), 39.3 (s, *C*H_2_), 39.1 (s, *C*H_2_), 35.5 (s, *C*H_2_), 33.7 (s, *C*H_2_), 27.9 (s, *C*H_2_), 26.4 (s, *C*H_2_), 25.5 (s, *C*H_2_), 25.0 (s, *C*H_2_), 24.9 (s, *C*H_2_), 23.9 (s, *C*H_2_). IR (ATR): 3301, 2928 (OH), 2865 (OH), 1709 (CO), 1639 (CONH), 1614, 1516, 1382, 1237, 1170, 940, 833 cm^−1^. HRMS: 281.1618 ([M + H]^+^, C_12_H_26_ClN_2_O_3_^+^; calc. 281.1626). A known compound, not described in the literature.

*4-((2-Aminoacetamido)methyl)benzoic acid hydrochloride* (**6**)

White solid, mp 235–238 °C. Yield 93% (0.226 g). ^1^H-NMR (D_2_O, 700 MHz): *δ* = 8.01 (d, *J*_HH_ = 8.2 Hz, 2 H, 2 × C*H*_Ar_), 7.46 (d, *J*_HH_ = 8.1 Hz, 2 H, 2 × C*H*_Ar_), 4.55 (s, 2 H, C*H*_2_), 3.93 (s, 2 H, C*H*_2_). ^13^C-NMR (D_2_O, 176 MHz): *δ* = 170.4 (s, *C*O), 167.0 (s, *C*O), 143.4 (s, *C*_Ar_), 130.0 (s, 2 × *C*_Ar_H), 128.8 (s, *C*_Ar_), 127.3 (s, 2 × *C*_Ar_H), 42.9 (s, *C*H_2_), 40.5 (s, *C*H_2_). IR (ATR): 3237, 2959 (OH), 2057 (Ar), 1662 (CO), 1600 (CONH), 1548, 1475, 1424, 1318, 1288, 1255, 1218,1103, 863, 532 cm^−1^. HRMS: 245.0682 ([M + H]^+^, C_10_H_14_ClN_2_O_3_^+^; calc. 245.0687). New compound.

*2-((((1s,4s)-4-Carboxycyclohexyl)methyl)amino)-2-oxoethanaminium chloride* (**7**)

White solid, mp 221–224 °C. Yield 94% (0.235 g). ^1^H-NMR (D_2_O, 700 MHz): *δ* = 3.83 (s, 2 H, C*H*_2_), 3.16 (d, *J*_HH_ = 6.7 Hz, 2 H, C*H*_2_), 2.36 (tt, *J*_HH_ = 12.3 Hz, *J*_HH_ = 3.4 Hz, 1 H, C*H*), 2.03–2.02 (m, 2 H, C*H*_2_), 1.85–1.83 (m, 2 H, C*H*_2_), 1.58–1.52 (m, 1 H, C*H*), 1.42 (qd, *J*_HH_ = 12.9 Hz, *J*_HH_ = 3.2 Hz, 2 H, C*H*_2_), 1.05 (qd, *J*_HH_ = 13.0Hz, *J*_HH_ = 3.2 Hz, 2 H, C*H*_2_). ^13^C-NMR (D_2_O, 176 MHz): *δ* = 181.4 (s, *C*O), 166.8 (s, *C*O), 45.4 (s, *C*H_2_), 42.9 (s, *C*H), 40.4 (s, *C*H_2_), 36.5 (s, *C*H), 28.9 (s, 2 × *C*H_2_), 28.1 (s, 2 × *C*H_2_). IR (ATR): 3284, 3021 (OH), 2923 (OH), 1690 (CO), 1655 (CONH), 1582, 1481, 1224, 1193, 1128, 828, 728 cm^−1^. HRMS: 251.1151 ([M + H]^+^, C_10_H_20_ClN_2_O_3_^+^; calc. 251.1157). New compound.

*6-(2-Aminoacetamido)hexanoic acid hydrochloride* (**8**)

White solid, mp 120–123 °C. Yield 90% (0.201 g). ^1^H-NMR (D_2_O, 700 MHz): *δ* = 3.82 (s, 2 H, C*H*_2_), 3.29 (t, *J*_HH_ = 6.9 Hz, 2 H, C*H*_2_), 2.43 (t, *J*_HH_ = 7.4 Hz, 2 H, C*H*_2_), 1.65 (qw, *J*_HH_ = 7.5 Hz, 2 H, C*H*_2_), 1.58 (qw, *J*_HH_ = 7.1 Hz, 2 H, C*H*_2_), 1.39 (qw, *J*_HH_ = 7.7 Hz, 2 H, C*H*_2_). ^13^C-NMR (D_2_O, 176 MHz): *δ* = 179.0 (s, *C*O), 166.6 (s, *C*O), 40.4 (s, *C*H_2_), 39.3 (s, *C*H_2_), 33.6 (s, *C*H_2_), 27.8 (s, *C*H_2_), 25.5 (s, *C*H_2_), 23.8 (s, *C*H_2_). IR (ATR): 3263, 3012 (OH), 2938 (OH), 1678 (CO), 1567 (CONH), 1478, 1388, 1256, 1224, 1166, 910, 829, 695 cm^−1^. HRMS: 225.1009 ([M + H]^+^, C_8_H_18_ClN_2_O_3_^+^; calc. 225.1000). New compound.

### 3.4. Evaluation of the Biological Activity of the Synthesized Compounds

#### 3.4.1. Hemolysis Assay 

Heparinized whole blood was used from a healthy volunteer (male, 37 years old). The whole blood (10 mL) was diluted with saline solution (90 mL). For each UV-Vis measurement, 2.5 mL of diluted blood was used. For each blood sample, 1 mL of a saline solution containing hydrochlorides **1**–**8** at concentrations of 5, 50, and 500 mg/L was added. As a control were used blood samples with the addition of 1% solution of DMSO in saline and 1% solution of SDS in saline. In all cases, the measurements were repeated twice for each concentration. The samples of blood were incubated for 45 min at 37 °C. The cells were centrifuged, and the absorbance of the supernatant, which contained plasma and lysed erythrocytes, was measured at 540 nm. The percentage of lysis was calculated from a standard curve of lysed erythrocytes treated with SDS [52].

#### 3.4.2. Blood Plasma Clotting Assays

Human blood plasma was isolated from fresh buffy coats (collected onto the citrate-dextrose solution—ACD) purchased from the Regional Centre of Blood Donation and Blood Treatment in Lodz, Poland. In the isolation procedure, the buffy coats (60 mL units) were divided into volumes of 10 mL and centrifuged (4000 rpm/15 min) in 15 mL tubes. Next, 1 mL solutions of each amide **1**–**8** were prepared in distilled water at concentrations of 10, 25, and 50 mg/L. Then, 450 μL of plasma and 50 μL of each amide at the tested concentrations were placed into 1.5 mL vials. The vials were vortexed (Vortex Biosan V-1 plus), incubated at 37 °C on a thermoblock (Dry Block Thermostat Biosan TDB-120), and analyzed using a Kselmed K-3002 OPTIC coagulometer (Grudziądz, Poland), based on the manufacturer’s protocols (described below). The control sample was native blood plasma, untreated with any of the examined substances.

#### 3.4.3. Prothrombin Time (PT)

Fifty microliters of control plasma and plasma samples treated with the amides were transferred to measuring cuvettes and placed in the thermoblock module (37 °C) of a coagulometer (KSELMED, K-3002 Optic). Then, 100 μL of Dia-PT reagent (DIAGON Kft. Hungary; cat. no. 81100) was added. Measurements were started immediately.

#### 3.4.4. Activated Partial Thromboplastin Time (aPTT)

Fifty microliters of control plasma and plasma samples treated with the amides were transferred to measuring cuvettes and placed in the thermoblock module (37 °C) of the coagulometer (KSELMED, K-3002 Optic). Then, 50 μL of Dia-APTT reagent (DIAGON Kft. Hungary; cat. no. 72048) was added. The mixture was incubated for 180 s at 37 °C. Finally, 50 μL of reagent Dia-CaCl_2_ (DIAGON Kft. Hungary; cat. no. 41192) was added. Measurements were started immediately.

#### 3.4.5. Thrombin Time (TT)

Both 100 μL of control plasma and plasma samples treated with the amides were transferred to measuring cuvettes, which were placed in the thermoblock module (37 °C) of the coagulometer (KSELMED K-3002 Optic). Then, 100 μL of thrombin solution (4.5 U/mL, in 0.9% NaCl; Biomed, Lublin, Poland) was added to the cuvette. Measurements were started immediately.

#### 3.4.6. Clot Formation and Fibrinolysis (CFF) Assay 

Thrombin was purchased from BioMed (Lublin, Poland). A tissue-type plasminogen activator (Actilyse^®^) was purchased from Boehringer Ingelheim, Ingelheim am Rhein, Germany. Kinetic measurements were executed using a SpectrostarNano microplate spectrophotometer (BMG LabTech, Ortenberg, Germany).

Measurements were performed using a kinetic model, as described in our previous work [53]. Briefly, blood plasma was preincubated with the examined substances (at final concentrations of 10, 25, and 50 mg/L) for 15 min at 37 °C. Then, 100 μL of blood plasma was added to microtiter plate wells, which was followed by 200 μL of the reagent mixture (0.75 U/mL thrombin, 225 ng/mL t-PA and 7.5 mM CaCl_2_) suspended in tris-buffered saline (0.05 M TBS, 0.9% NaCl; pH 7.4). Measurements of absorbance changes were started immediately and continued for 60 min at 37 °C, λ = 360 nm. The following parameters were used to determine the effects of the examined substances on the coagulation and fibrinolytic properties of the blood plasma: the maximal velocity of fibrinogen polymerization (V_maxC_), which is an indicator of the plasma coagulation efficiency; the maximal absorbance (A_max_), which is an indicator of the fibrin clot stabilization and its thickness; the maximal velocity of clot lysis (V_maxF_), which is an indicator of blood plasma fibrinolytic activity.

#### 3.4.7. Cell Culture

All experiments were conducted on the commercially available monocyte/macrophage peripheral blood cell line SC (ATCC CRL-9855) (ATCC; Manassas, VA, USA). The cell line was maintained under standard conditions, according to the guidelines provided by the vendors (37 °C, 5% CO_2_, 95% humidity). The SC cells were cultured in Iscove’s Modified Dulbecco’s Medium (IMDM; ATCC; Manassas, VA, USA) with 4 mM L-glutamine and 1.5 g/L sodium bicarbonate, which was supplemented with 0.05 mM 2-mercaptoethanol (Sigma-Aldrich Corp., St. Louis, MO, USA), 0.1 mM hypoxanthine, 0.016 mM thymidine (90%) (ATCC; Manassas, VA, USA), 10% fetal bovine serum (ATCC; Manassas, VA, USA), and 1% penicillin/streptomycin solution (P/S) (ScienCell Research Laboratories, San Diego ad, CA, USA).

#### 3.4.8. Cytotoxicity Analysis 

Cytotoxicity analysis was performed using the colorimetric 2,3-bis-(2-methoxy-4-nitro-5-sulfophenyl)-2H-tetrazolium-5-carboxanilide (XTT) assays (Thermo Scientific, Waltham, MA, USA), which enables assessment of cell viability as a function of redox potential. All the experiments were performed in triplicate with similar results. The cells were seeded and incubated in 96-well plates (7 × 10^3^ cells per well). To assess the cytotoxicity of the investigated compounds, dilutions were prepared in the range from 50 to 1.5 μM. Untreated cells cultured in a complete medium were used as a negative control. Cells incubated with 100% DMSO (Sigma-Aldrich Corp., St. Louis, MO, USA) were used as a positive control. After 48 h of incubation, 25 µL of XTT/PMS mixture was added to each well. After 4 h of incubation at 37 °C in a 5% CO_2_ incubator, absorbance was measured at a wavelength of 450 nm using a spectrophotometer (Synergy HT, BioTek, Winooski, VT 05404, USA).

#### 3.4.9. Genotoxicity Analysis

Genotoxicity was assessed using the alkaline version of the comet assay, which is commonly used to determine the level of DNA damage at the level of individual cells. The alkaline version of the comet assay enables the assessment of oxidative damage, single-stranded cracks, double-stranded cracks, and alkaline labile sites. All of the experiments were performed in triplicate with similar results. SC cells were seeded and incubated in 12-well plates (2 × 10^5^/well) and cultured in 1 mL of complete IMDM medium for 24 h. Untreated cells were used as a negative control. Cells incubated with 10% DMSO (Sigma-Aldrich Corp., St. Louis, MO, USA) were used as a positive control. After 48 h of incubation with the investigated compounds, the SC cells were centrifuged and suspended in 0.37% LMP (low melting point) agarose (Sigma-Aldrich Corp., St. Louis, MO, USA). Subsequently, a suspension of the cells in 0.37% LMP agarose was placed on microscope slides coated with NMP (normal melting point) agarose (Sigma-Aldrich Corp., St. Louis, MO, USA). The obtained preparations were incubated in lysis buffer at pH 10 (2.5-M NaCl, 10-mM Tris, 100-mM EDTA) with 1% X-100 Triton (Sigma-Aldrich Corp., St. Louis, MO, USA) for 1 h at 4 °C. After lysis, the cells were incubated with development buffer (300 mM NaOH, 1 mM EDTA) for 20 min at 4 °C. Then, the preparations were electrophoresed (32 mA, 17 V, 20 min) at 4 °C, in electrophoretic buffer (30 mM NaOH, 1 mM EDTA). The slides were rinsed three times with distilled water and allowed to dry completely at RT. After staining with a fluorescent dye (DAPI), the preparations were analyzed under a fluorescent microscope. The amount of DNA damage in the cells was measured as the percentage of DNA in the comet tail.

#### 3.4.10. Statistical Analysis

Statistical analysis was performed using the Sigma Plot program (Systat Software, Inc., San Jose, CA, USA). The Shapiro–Wilk test was used to perform a normality test for the statistical analysis of cell viability and the comet assay. The cell viability analyses were normally distributed. Therefore, statistical analysis of the two groups was performed using the Student’s t-test. In the comet assay, no normal distribution was obtained. Therefore, statistical analysis of the two groups was performed using the Mann–Whitney rank-sum test. Each of the statistical analyses was based on the results of three independent tests. Statistical significance in the CFF assay was established using the Wilcoxon test. In the figures and tables, the differences are statistically significant as follows: * *p* < 0.05, ** *p* < 0.01, *** *p* < 0.001.

## 4. Conclusions

Eight hydrochloride dipeptides **1**–**8** containing antifibrinolytic agents (tranexamic acid, aminocaproic acid, and 4-(aminomethyl)benzoic acid) and a natural amino acid (glycine) were synthesized with very good yields, using DMT/NMM/TsO^−^ (4-(4,6-dimethoxy-1,3,5-triazin-2-yl)-4-methylmorpholinium toluene-4-sulfonate) as a coupling reagent. The compounds were tested for their hemostatic properties and submitted to blood plasma clotting tests. None of the synthesized compounds **1**–**8** caused hemolysis at any concentration. The level of hemolysis did not exceed 1%. In clotting tests, thrombin time (TT), prothrombin time (PT), and activated partial thromboplastin time (aPTT) did not differentiate any of the compounds. The prothrombin times for all amides **1**–**8** were similar. However, the results for amides **1**–**4** and **8** were slightly lower than for the other compounds and the positive control. They were also similar to the results obtained for TA. The activity of the other compounds did not statistically affect aPTT except in the case of amide **3**, which significantly decreased aPTT. The aPTTs recorded for plasma treated with amide **3** and TA were similar. Thrombin time (TT) for amides **1**–**8** was mostly comparable to the control sample. Only in the case of amide **6** and **8** was TT significantly lower than for the other compounds. In the clot formation and fibrinolysis (CFF) assay, amides **5** and **7** were among the most active compounds. The maximal velocity of fibrin polymerization (V_maxC_) and the maximal absorbance (A_max_) of the fibrin clot indicate that among the tested compounds, amide **5** had the most evident procoagulant activity. Measurements of the maximal velocity of clot lysis (V_maxF_) indicate that amides **4** and **7** were the most active inhibitors of the fibrinolytic activity of blood plasma. The synthesized amides **1**–**8** did not exhibit significant cytotoxic effects toward SC cells and did not induce significant DNA damage. The results of hemostatic properties of amides **1**–**8** (hemolysis, blood plasma clotting assays: PT, aPTT, TT) are summarized in Table 2.

Research on the design and synthesis of new lysine analogs with hemostatic activity will be continued.

## Figures and Tables

**Table 1:** Effects of the examined amides on the hemostatic properties of human blood plasma. The effects of each type of amide were determined in the CFF assay, based on the maximal velocity of fibrin polymerization/clotting (V_maxC_), the maximum of fibrin clot absorbance at 360 nm (A_max_), and the maximal velocity of fibrin clot lysis (V_maxF_). The hemostatic activity of the control plasma was assumed as 100% (of V_maxC_, V_maxF_, and A_max_). The results are presented as the mean ± SD; *n* = number of independent experiments, * *p* < 0.05, ** *p* < 0.001; *** *p* < 0.001; *n* = 5–7.

Type of Sample	Concentration [mg/L]	V_maxC_ (of Fibrin Polymerization)	A_max_	V_maxF_ (of Fibrin Clot Lysis)
**Control Plasma**	0	100.00 %	100.00%	100.00%
**1**	10	106.12 ± 4.28 **	105.98 ± 4.31 *	99.08 ± 9.93
	25	105.66 ± 6.27	107.24 ± 8.91	87.32 ± 20.27
	50	104.20 ± 3.85 *	104.62 ± 9.46	95.58 ± 15.97
**2**	10	103.26 ± 3.98	105.29 ± 6.41	75.81 ± 17.04 *
	25	106.30 ± 3.60 **	110.74 ± 4.86 *	79.60 ± 10.55 *
	50	105.59 ± 3.74 **	110.77 ± 9.85 *	85.69 ± 8.80 *
**3**	10	105.61 ± 7.06	107.21 ± 6.90 *	93.23 ± 16.83
	25	103.88 ± 9.73	104.49 ± 9.80	97.05 ± 24.39
	50	109.78 ± 6.46 *	112.98 ± 8.40 *	102.82 ± 20.01
**4**	10	107.93 ± 7.23 *	115.37 ± 8.73 *	81.36 ± 21.07 *
	25	105.85 ± 7.85	114.07 ± 15.71	70.35 ± 16.30 *
	50	105.89 ± 5.64	109.29 ± 9.02 *	55.95 ± 10.51 *
**5**	10	105.80 ± 2.97 **	106.44 ± 1.78 *	90.08 ± 13.68
	25	105.88 ± 3.76 **	107.12 ± 4.65 *	85.12 ± 16.98 *
	50	109.83 ± 1.46 ***	113.43 ± 7.86 *	89.46 ± 10.48 *
**6**	10	104.68 ± 3.05 *	105.45 ± 5.46	98.41 ± 14.71
	25	103.10 ± 6.82	102.41 ± 5.56	100.06 ± 21.96
	50	101.11 ± 4.77	102.15 ± 2.46	91.08 ± 15.49
**7**	10	106.86 ± 9.49	115.64 ± 11.61 *	66.18 ± 21.32 *
	25	104.39 ± 8.60	120.06 ± 25.63 *	62.99 ± 16.99 *
	50	111.45 ± 8.07 **	113.54 ± 13.76 *	87.27 ± 8.04
**8**	10	101.96 ± 6.62	104.83 ± 2.83 *	82.96 ± 12.49 *
	25	100.98 ± 6.60	100.18 ± 3.77	96.07 ± 14.56
	50	105.67 ± 8.34	108.64 ± 11.94	66.90 ± 23.22 *
**TA**	10	101.38 ± 7.89	106.67 ± 3.79 *	total inhibition
	25	104.75 ± 9.98	105.87 ± 6.23	total inhibition
	50	104.85 ± 12.06	106.64 ± 4.65 *	total inhibition

**Table 2:** Summary of the hemolysis assay and blood plasma clotting assays for amides **1**–**8**.

Amide	Hemolysis Assay			Blood Plasma Clotting Assays			
	c [mg/L]	Time [min]	Hemolysis [%] *	c [mg/L]	PT Time [% of Control]	aPTT Time [% of Control]	TT Time [% of Control]
**1**	5	45	0.0	10	96.3 ± 2.5	108.3 ± 11.9	95.9 ± 6.3
		120	0.56				
		240	0.42				
	50	45	0.5	25	95.0 ± 2.9	105.1 ± 7.7	96.1 ± 6.8
		120	0.18				
		240	0.35				
	500	45	0.27	50	95.7 ± 2.2	101.9 ± 9.3	94.3 ± 8.3
		120	0.59				
		240	0.58				
**2**	5	45	0.41	10	97.0 ± 4.8	98.5 ± 8.0	99.0 ± 1.8
		120	0.21				
		240	0.65				
	50	45	0.11	25	96.1 ± 2.2	101.0 ± 13.1	98.9 ± 4.7
		120	0.74				
		240	0.30				
	500	45	1.03	50	95.5 ± 2.5	101.9 ± 14.0	95.9 ± 6.8
		120	0.21				
		240	0.43				
**3**	5	45	0.44	10	94.9 ± 2.2	94.8 ± 4.5	100.1 ± 2.6
		120	0.74				
		240	0.41				
	50	45	0.0	25	95.2 ± 3.0	94.7 ± 4.1	100.8 ± 6.9
		120	0.0				
		240	0.26				
	500	45	0.47	50	97.0 ± 3.6	96.3 ± 2.8	97.2 ± 4.3
		120	0.65				
		240	0.23				
**4**	5	45	1.0	10	97.3 ± 4.2	98.1 ± 5.2	96.3 ± 6.9
		120	0.74				
		240	0.27				
	50	45	0.0	25	95.4 ± 2.3	97.4 ± 5.4	95.5 ± 8.8
		120	0.71				
		240	0.32				
	500	45	0.38	50	93.7 ± 3.0	99.9 ± 6.0	97.6 ± 5.0
		120	0.21				
		240	0.22				
**5**	5	45	0.0	10	95.0 ± 10.5	98.8 ± 5.8	99.1 ± 6.1
		120	0.56				
		240	0.42				
	50	45	0.5	25	92.0 ± 8.5	101.1 ± 4.9	99.0 ± 4.7
		120	0.18				
		240	0.35				
	500	45	0.27	50	93.0 ± 8.3	100.8 ± 2.6	97.8 ± 3.3
		120	0.59				
		240	0.58				
**6**	5	45	0.38	10	96.1 ± 4.4	96.1 ± 4.2	92.5 ± 3.0
		120	0.56				
		240	0.19				
	50	45	0.0	25	95.8 ± 4.3	97.0 ± 3.5	92.3 ± 2.5
		120	0.11				
		240	0.2				
	500	45	0.41	50	93.5 ± 7.1	98.3 ± 3.6	92.8 ± 4.2
		120	0.68				
		240	0.31				
**7**	5	45	0.0	10	95.4 ± 7.4	98.1 ± 4.6	97.2 ± 3.3
		120	0.21				
		240	0.21				
	50	45	0.41	25	94.3 ± 9.7	99.3 ± 3.3	97.5 ± 2.5
		120	0.97				
		240	0.64				
	50	45	0.0	50	95.3 ± 8.3	100.6 ± 4.9	96.9 ± 1.7
		120	0.23				
		240	0.38				
**8**	5	45	0.08	10	93.0 ± 2.2	95.1 ± 7.5	100.0 ± 7.0
		120	0.74				
		240	0.36				
	50	45	0.44	25	95.5 ± 4.3	97.7 ± 5.9	94.3 ± 1.3
		120	0.71				
		240	0.3				
	50	45	0.0	50	97.2 ± 3.2	95.2 ± 7.3	92.4 ± 5.2
		120	0.47				
		240	0.37				

* Very low levels of hemolysis may cause the obtained results to fall within the measurement errors of the analysis.

## Data Availability

Not applicable.

## Supplementary Materials

The following supporting information can be downloaded at: https://www.mdpi.com/article/10.3390/molecules27072271/s1, Figures S1–S32, ^1^H and ^13^C-NMR spectra of compounds **1**–**8** and **18**–**25**; Figures S33–S40, graphs of hemolysis in the presence of compounds **1**–**8**; Figures S41–S43, graphs of prothrombin time, activated partial thromboplastin time, and thrombin time in the presence of compounds **1**–**8**.

## Sample Availability

Samples of the compounds **1**–**8** and **18**–**25** are available from the authors.
