# Supplementary material for: Synthesis and Hemostatic Activity of New Amide Derivatives
Source: Molecules. 2022 Mar 31;27(7):2271. doi: 10.3390/molecules27072271 (PMC9000710; doi:10.3390/molecules27072271)
Supplement: Supplementary file 1 [file molecules-27-02271-s001.zip › molecules-1656136-supplementary.pdf]

Synthesis and Hemostatic Activity of New Amide Derivatives

Lukasz Banach [1], Lukasz Janczewski [1], Jakub Kajdanek [2], Katarzyna Milowska [2], Joanna Kolodziejczyk-Czepas [3], Grzegorz Galita [4], Wioletta Rozpedek-Kaminska [4], Ewa Kucharska [5], Ireneusz Majsterek [4] and Beata Kolesinska [1,*]

[1] Faculty of Chemistry, Institute of Organic Chemistry, Lodz University of Technology, Zeromskiego 116, 90-924 Lodz, Poland; lukasz.banach@dokt.p.lodz.pl (L.B.); lukasz.janczewski@p.lodz.pl (L.J.)
[2] Department of General Biophysics, Faculty of Biology and Environmental Protection, University of Lodz, Pomorska 141/143, 90-236 Lodz, Poland; jakub.kajdanek@edu.uni.lodz.pl (J.K.); katarzyna.milowska@biol.uni.lodz.pl (K.M.)
[3] Department of General Biochemistry, Faculty of Biology and Environmental Protection, University of Lodz, Pomorska 141/143, 90-236 Lodz, Poland; joanna.kolodziejczyk@biol.uni.lodz.pl
[4] Department of Clinical Chemistry and Biochemistry, Medical University of Lodz, 90-419 Lodz, Poland; grzegorz.galita@umed.lodz.pl (G.G.); wioletta.rozpedek@umed.lodz.pl (W.R.-K.); ireneusz.majsterek@umed.lodz.pl (I.M.)
[5] Department of Gerontology, Geriatrics and Social Work, Jesuit University Ignatianum in Cracow, Kopernika 26, 31-501 Krakow, Poland; ewa.kucharska@ignatianum.edu.pl
* Correspondence: beata.kolesinska@p.lodz.pl; Tel.: +48-42-631-32-61

Table of contents:

Figures S1–S32: Copies of ^{1}H and ^{13}C NMR spectra of compounds **18–25** and **1–8** S2–S17

Figures S33–S40: Graphs of hemolysis in the presence of compounds **1–8** S18–S20

Figures S41–S43: Graphs of prothrombin time, activated partial thromboplastin time, and thrombin time in the presence of compounds **1–8** S21–S22

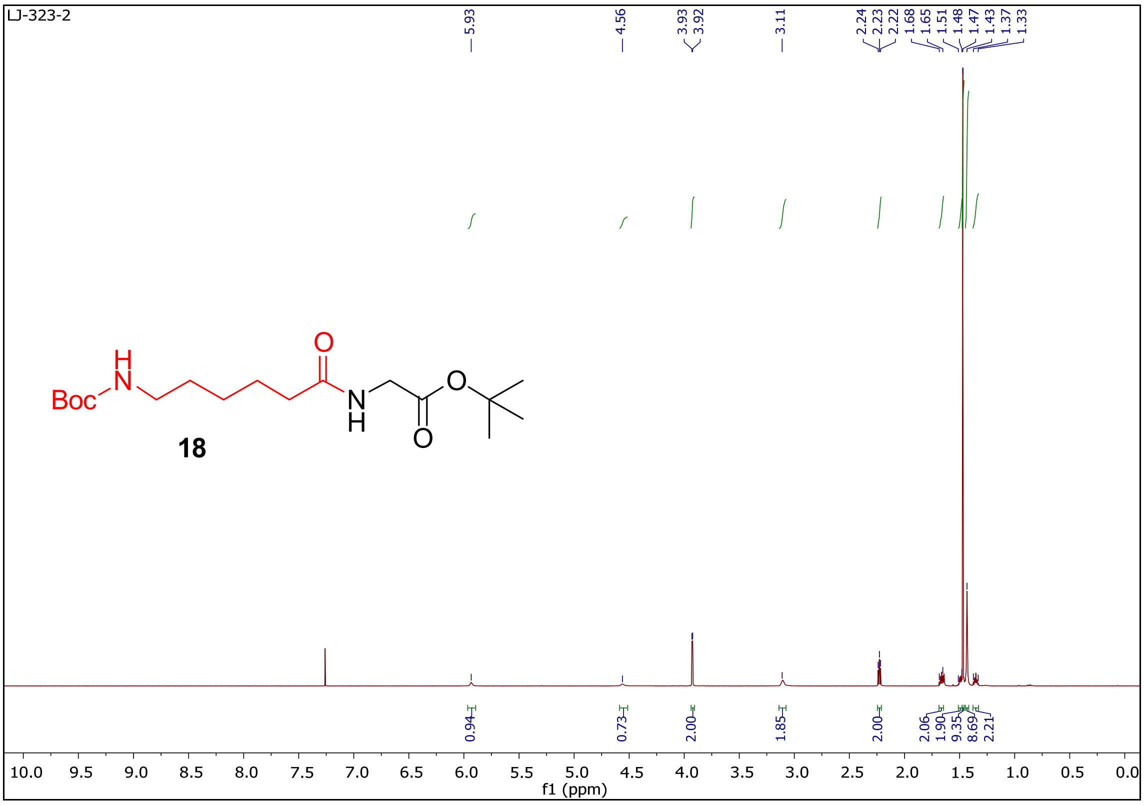

Figure S1. ^{1}H NMR of compound **18** (700 MHz, $CDCl_3$).

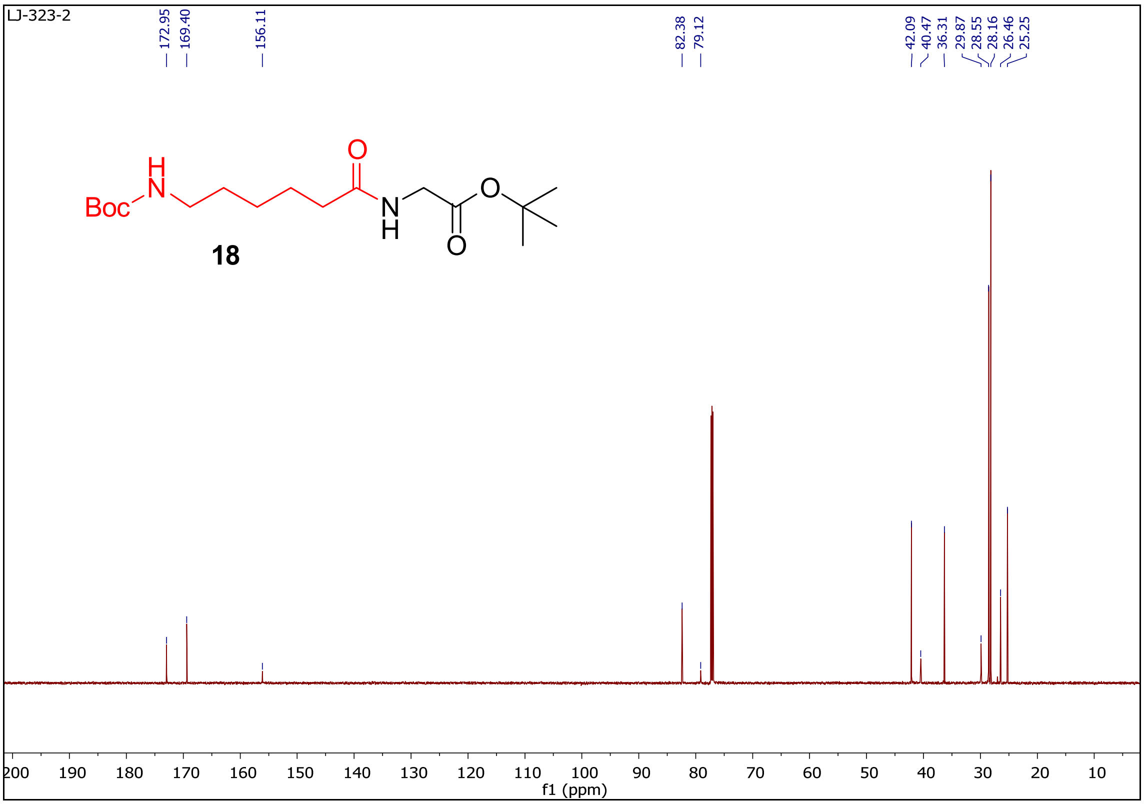

Figure S2. ^{13}C NMR of compound **18** (176 MHz, $CDCl_3$).

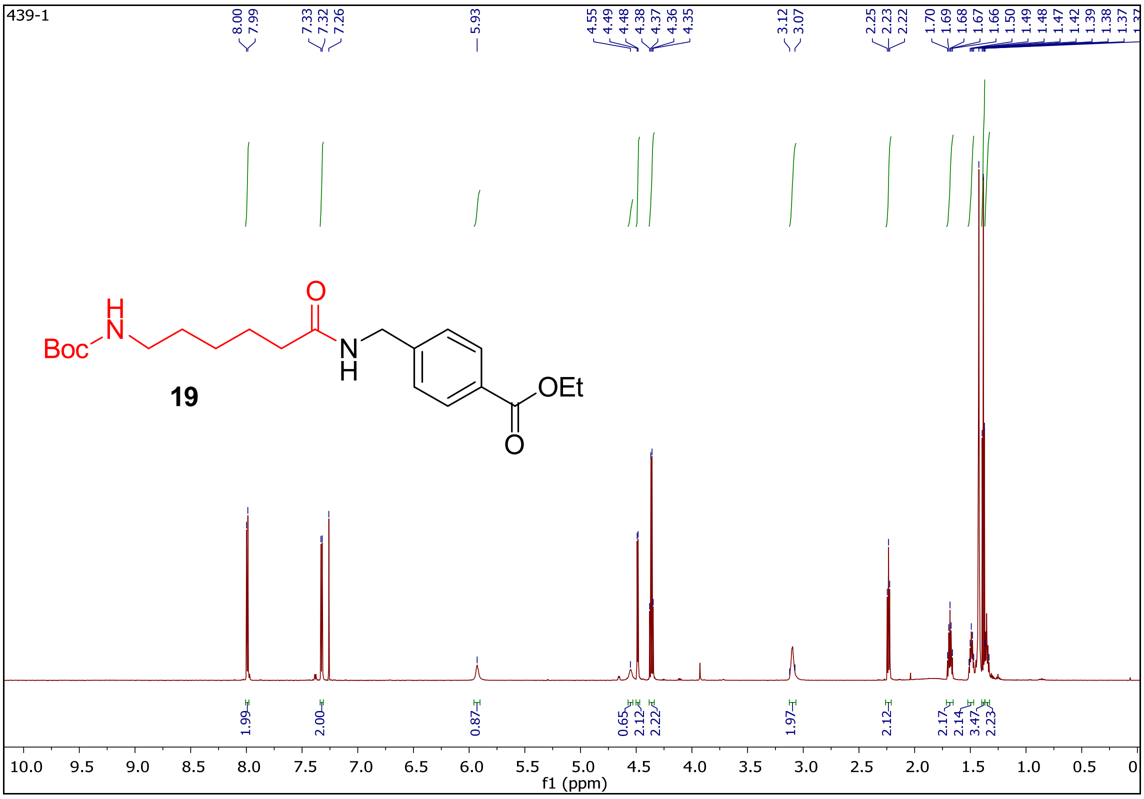

Figure S3. ^{1}H NMR of compound **19** (700 MHz, $CDCl_3$).

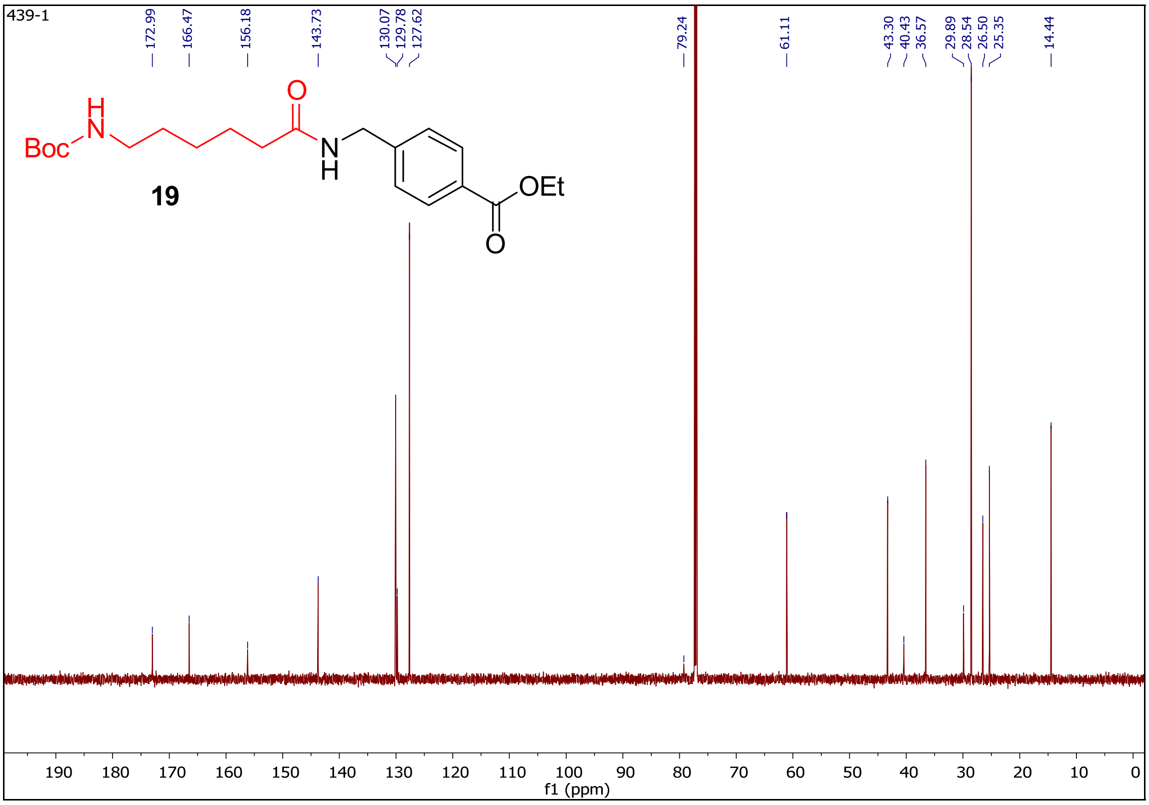

Figure S4. ^{13}C NMR of compound **19** (176 MHz, $CDCl_3$).

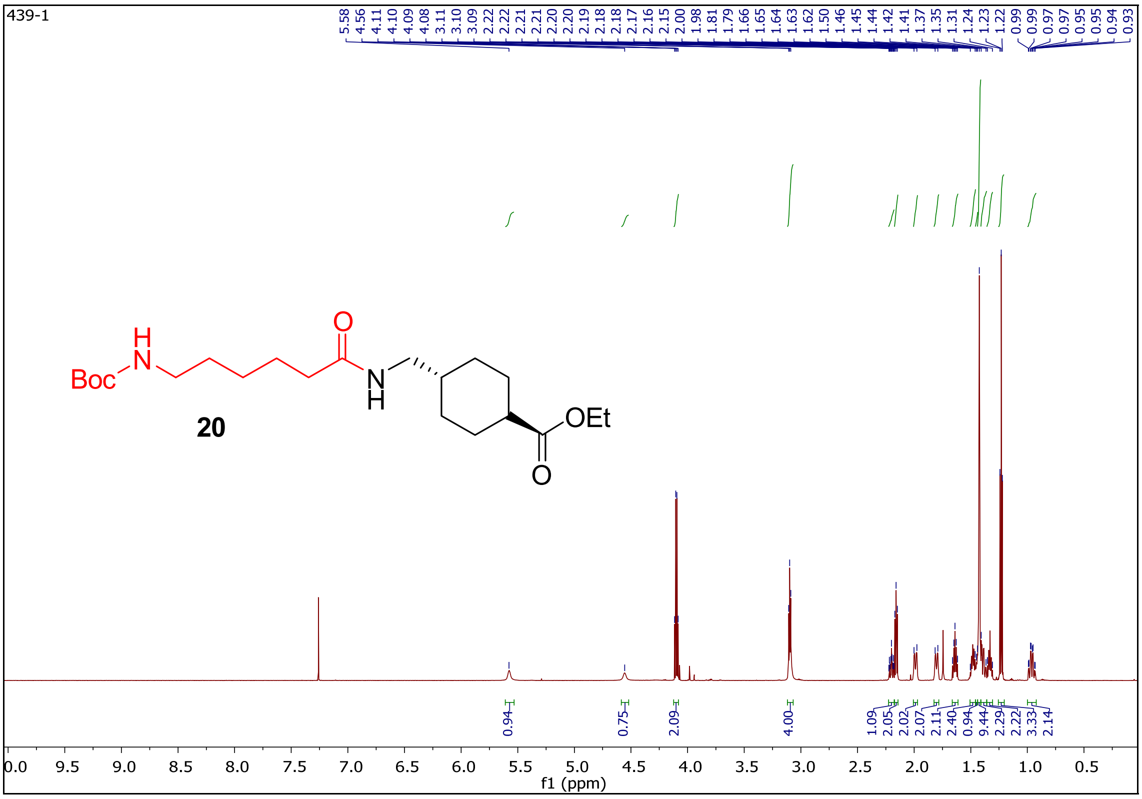

Figure S5. ^{1}H NMR of compound **20** (700 MHz, $CDCl_3$).

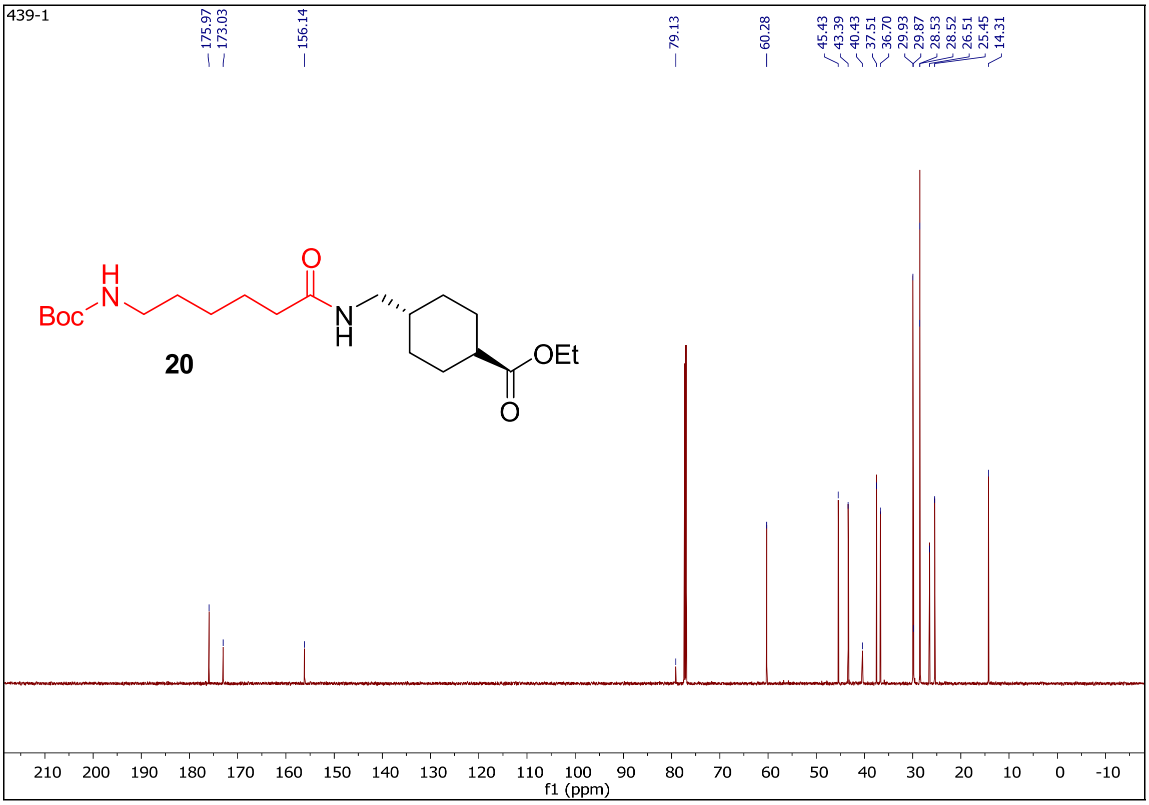

Figure S6. ^{13}C NMR of compound **20** (176 MHz, $CDCl_3$).

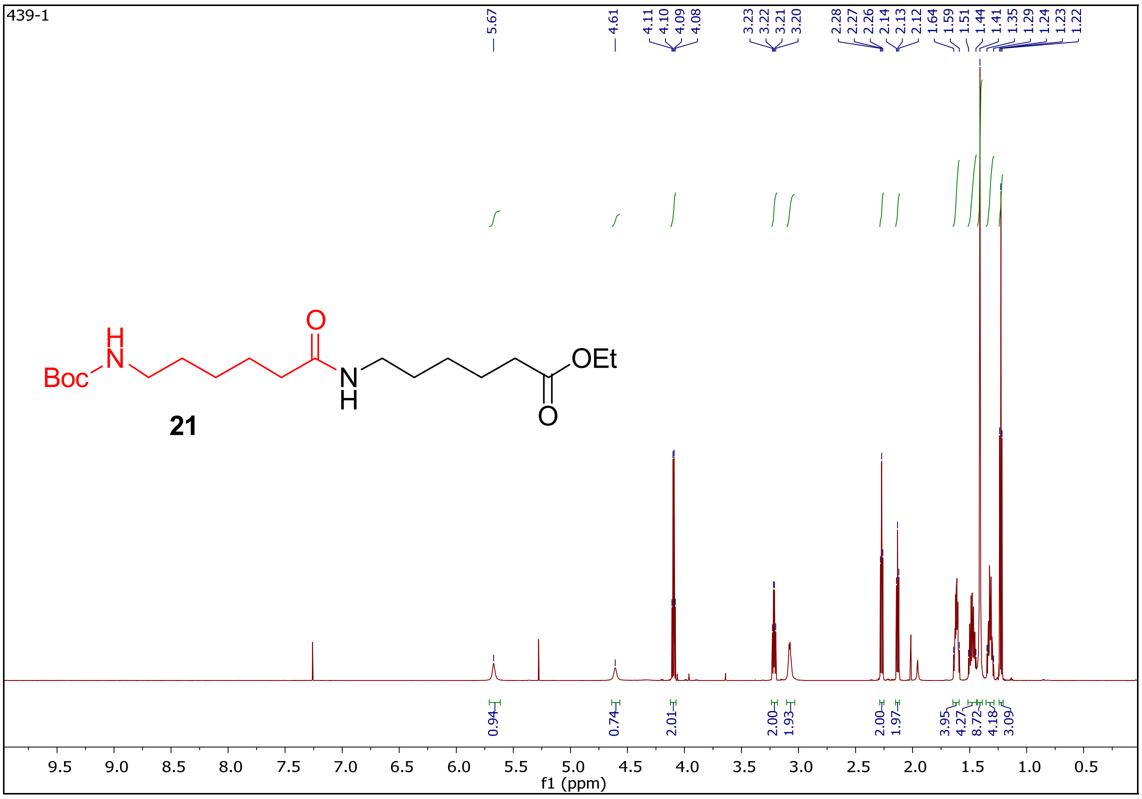

Figure S7. ^{1}H NMR of compound **21** (700 MHz, $CDCl_3$).

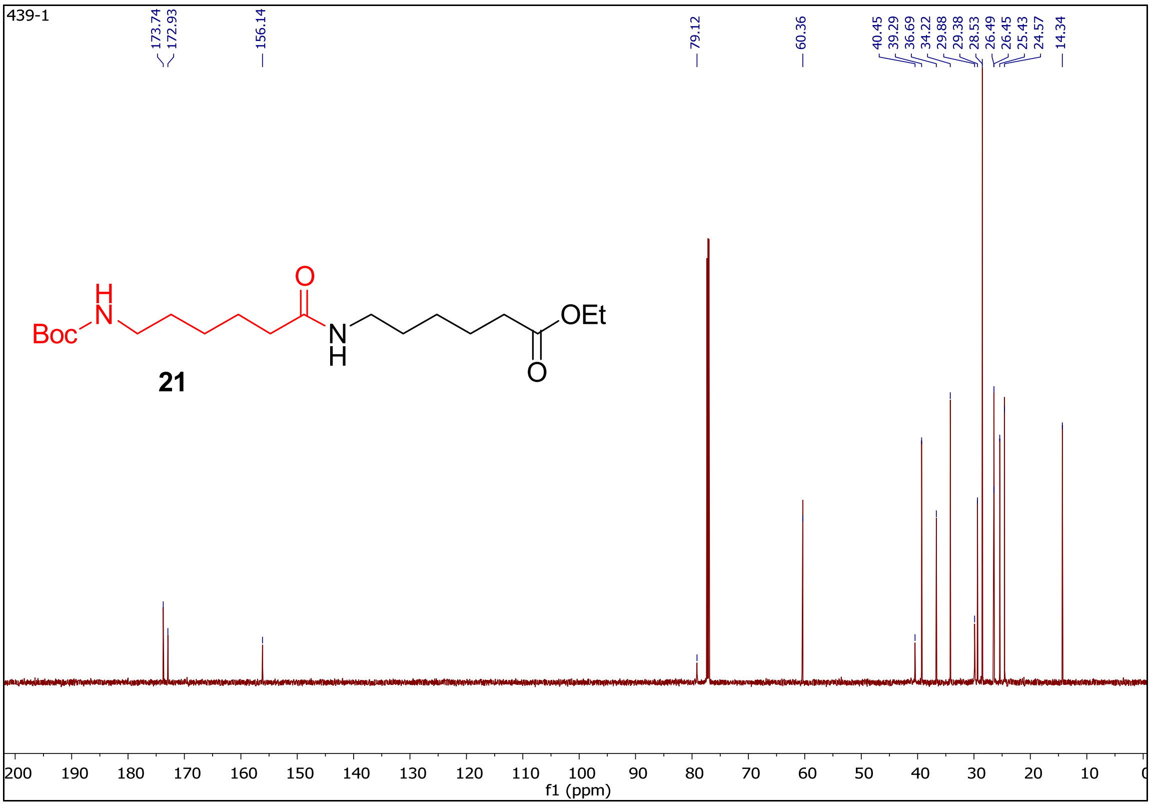

Figure S8. ^{13}C NMR of compound **21** (176 MHz, $CDCl_3$).

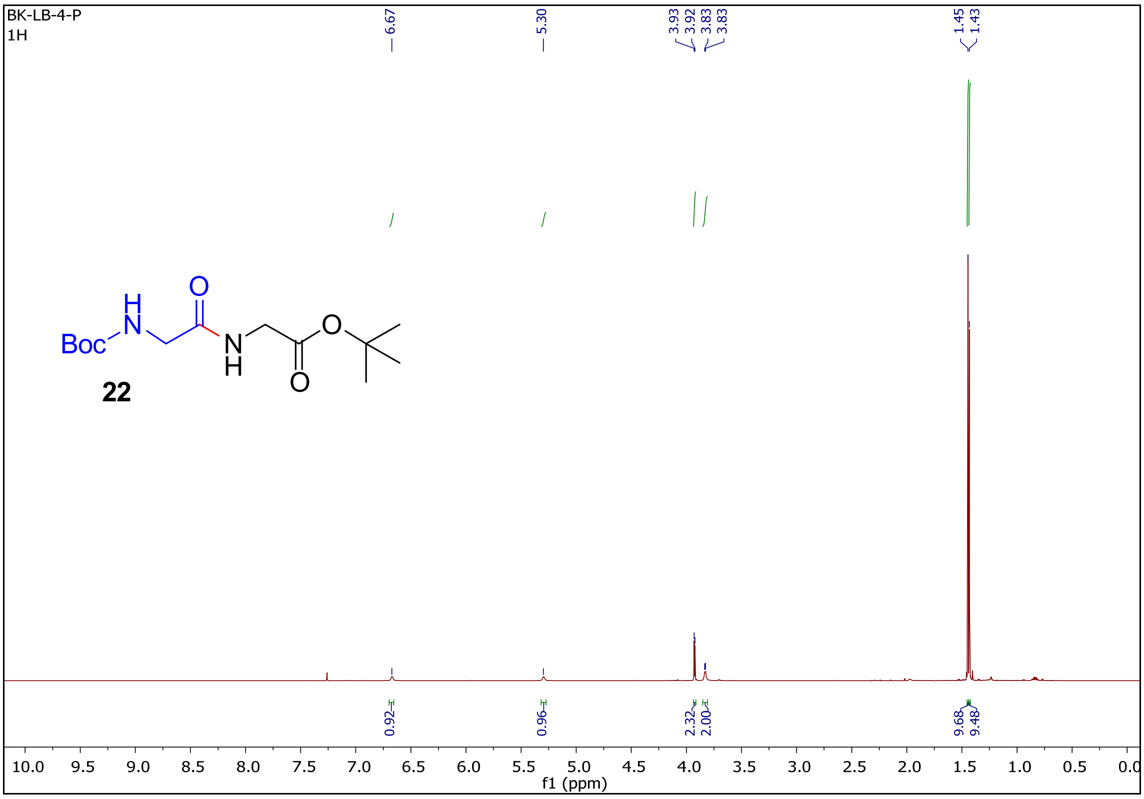

Figure S9. ^{1}H NMR of compound **22** (700 MHz, $CDCl_3$).

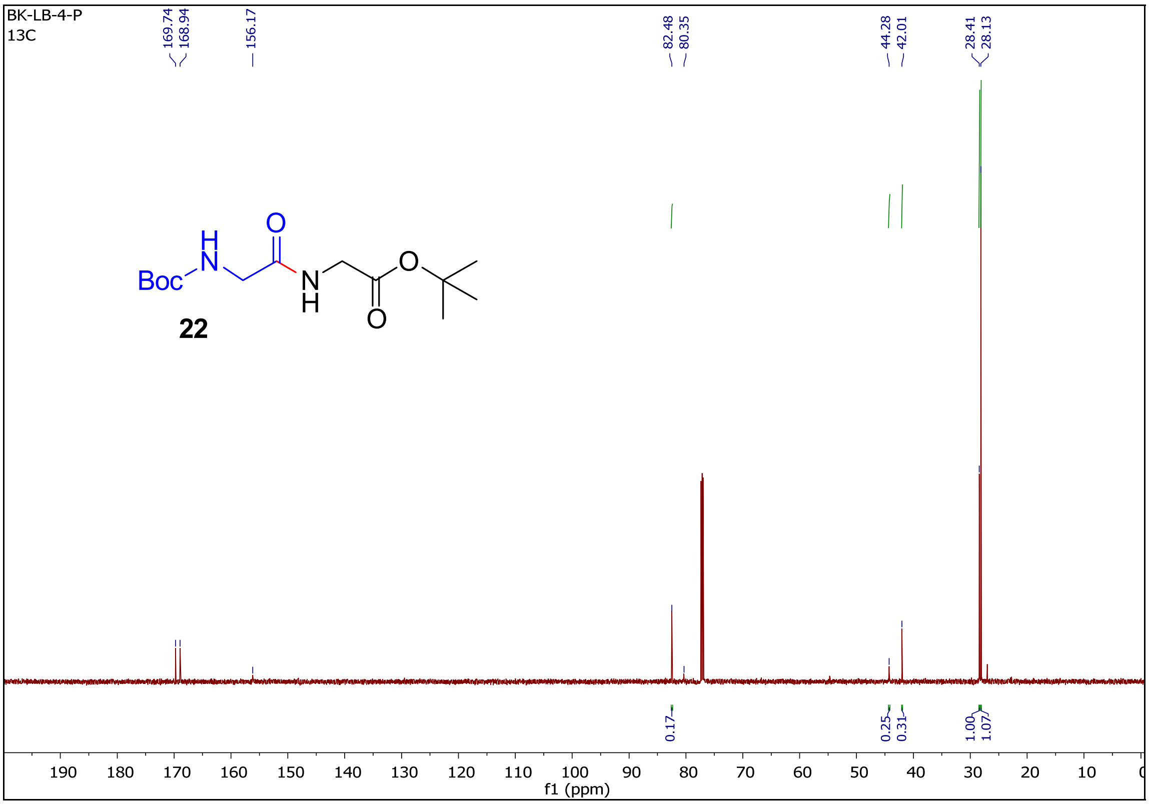

Figure S10. ^{13}C NMR of compound **22** (176 MHz, $CDCl_3$).

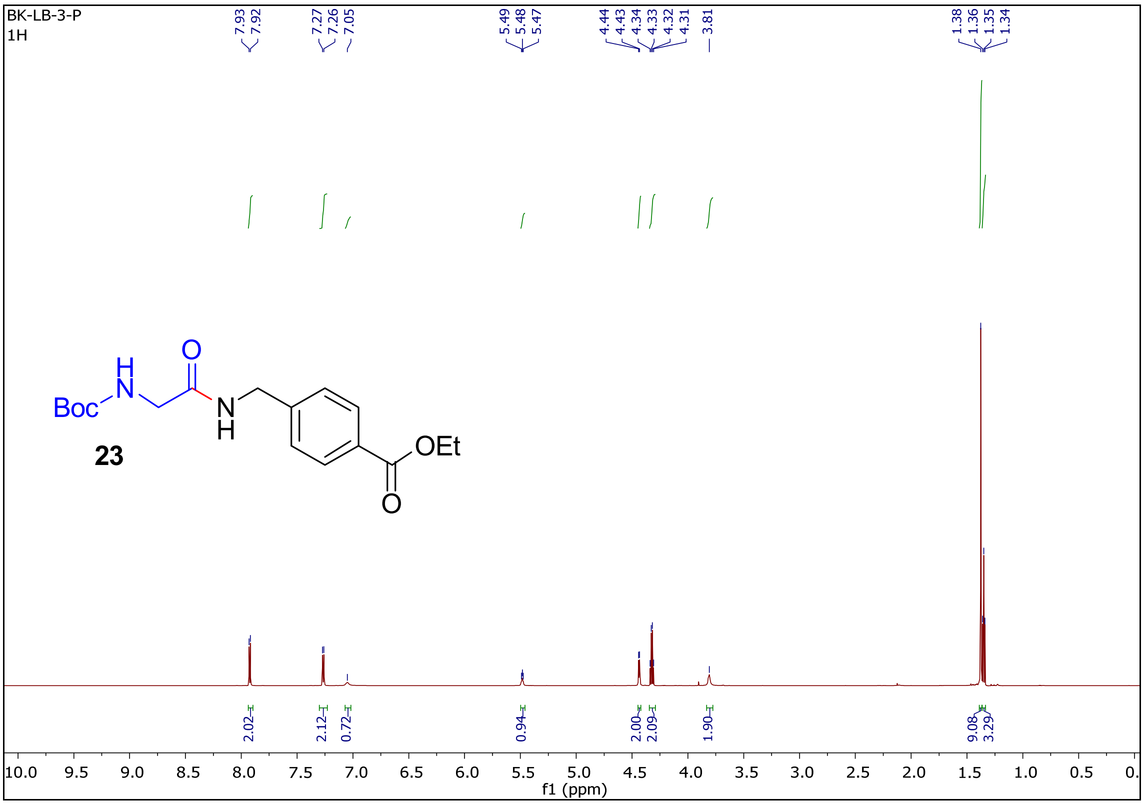

Figure S11. ^{1}H NMR of compound **23** (700 MHz, $CDCl_3$).

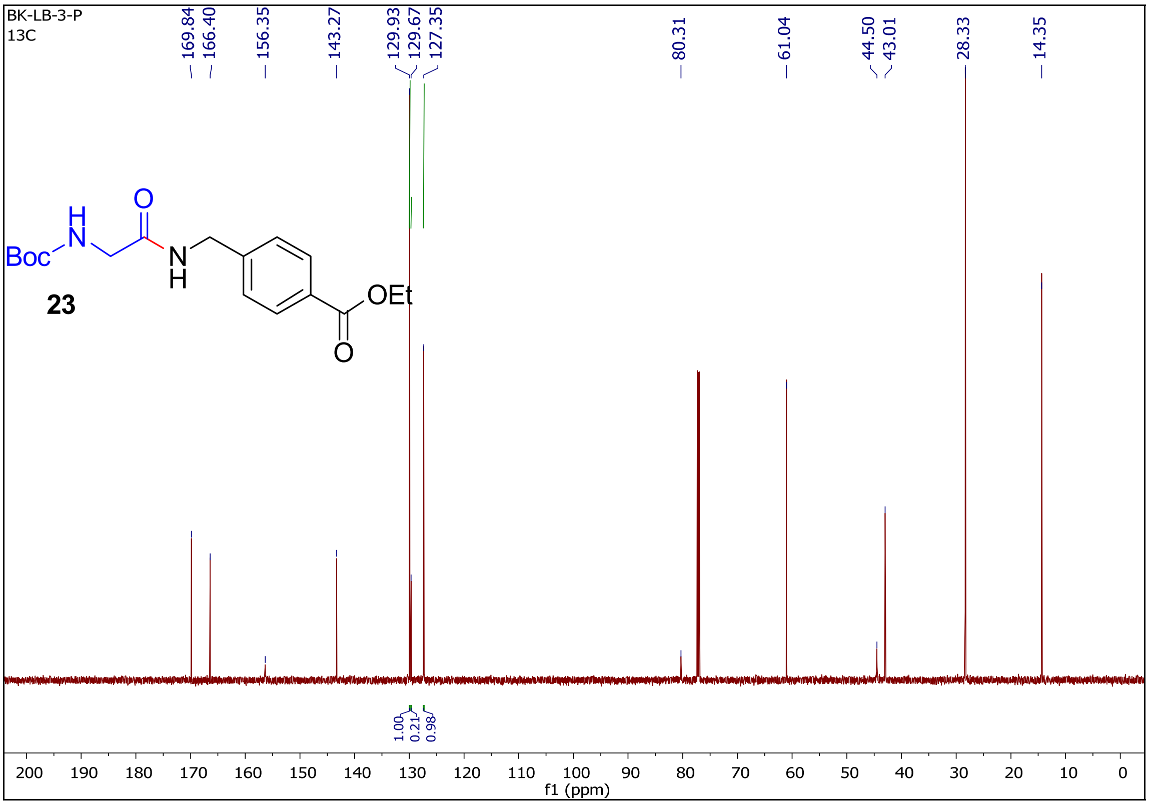

Figure S12. ^{1}H NMR of compound **23** (176 MHz, $CDCl_3$).

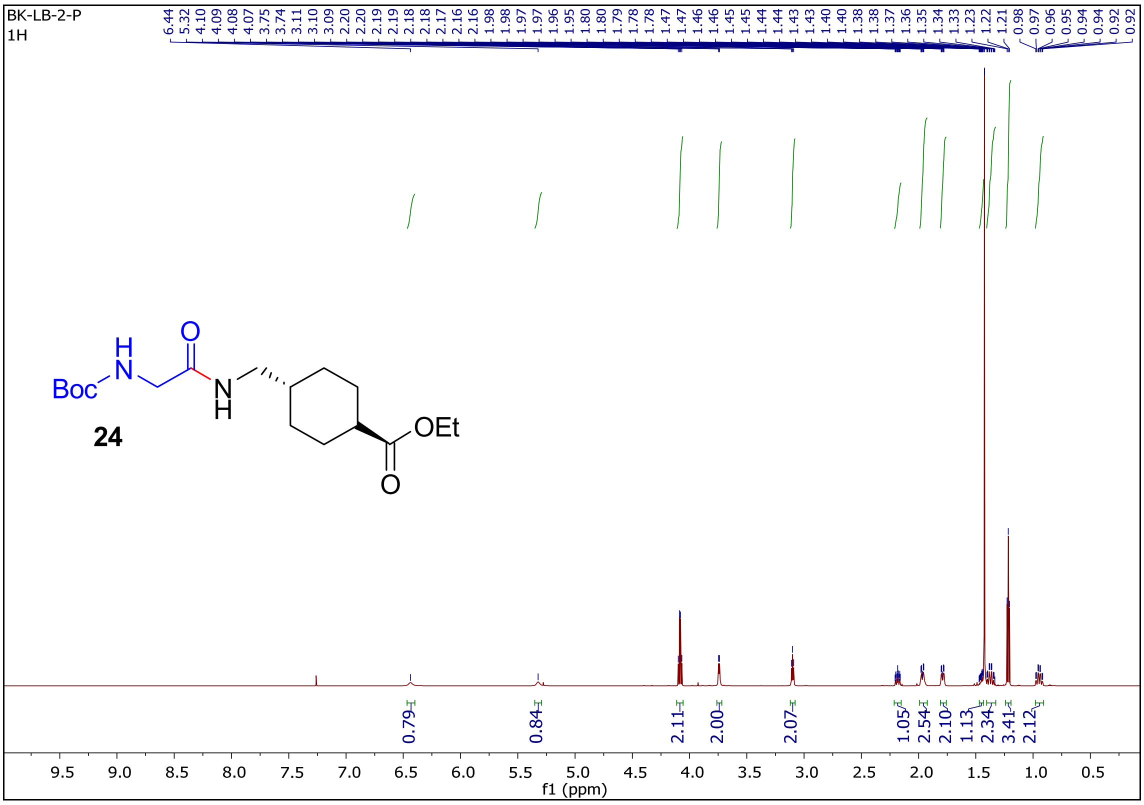

Figure S13. ^{1}H NMR of compound **24** (700 MHz, $CDCl_3$).

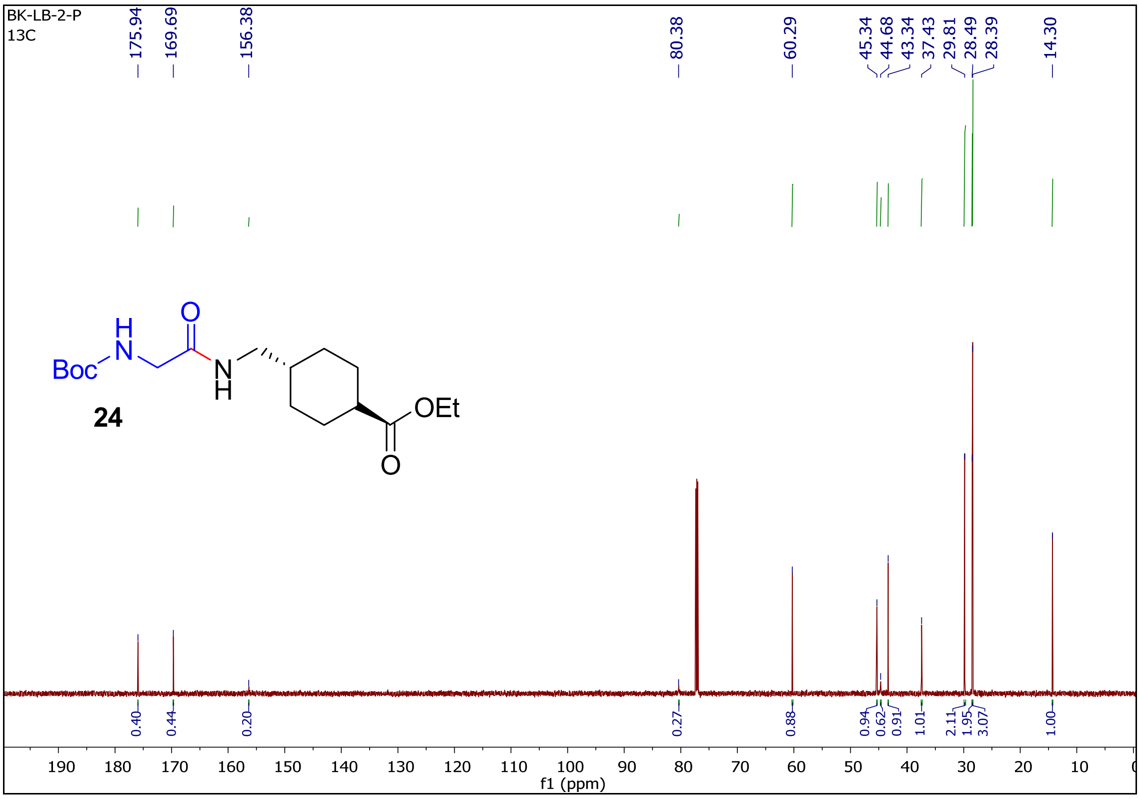

Figure S14. ^{13}C NMR of compound **24** (176 MHz, $CDCl_3$).

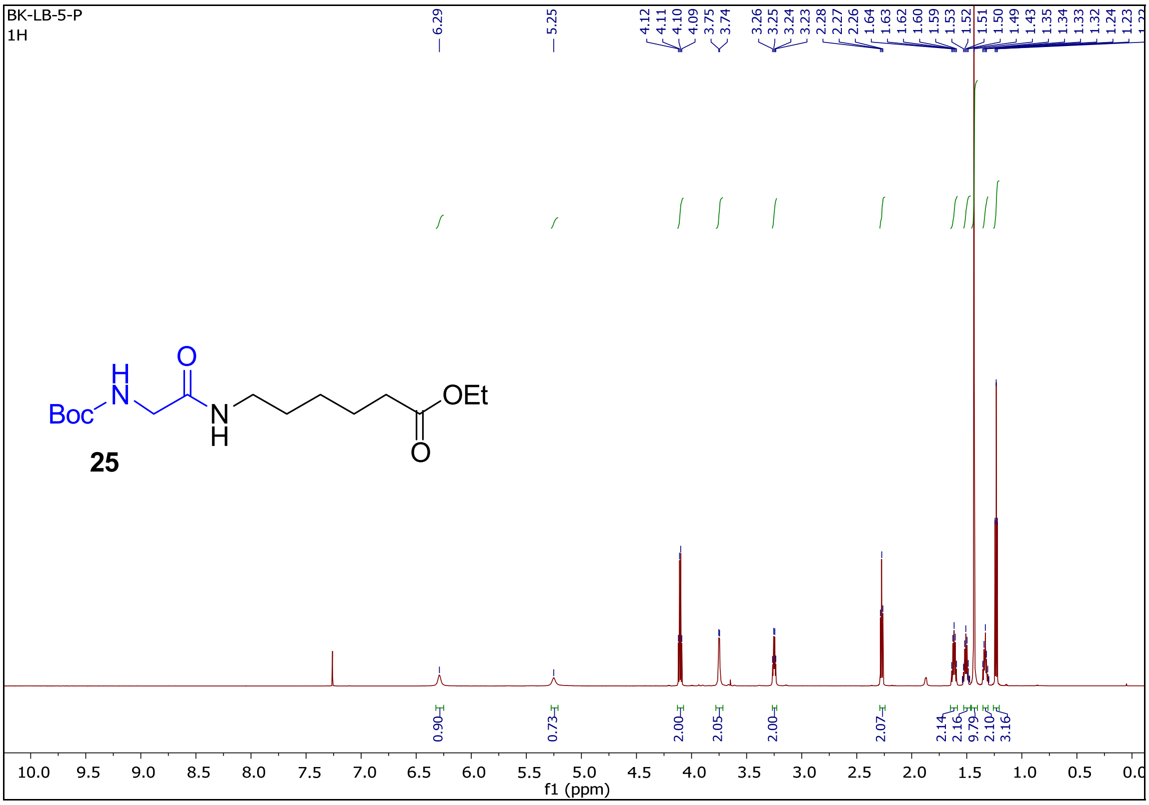

Figure S15. ^{1}H NMR of compound **25** (700 MHz, $CDCl_3$).

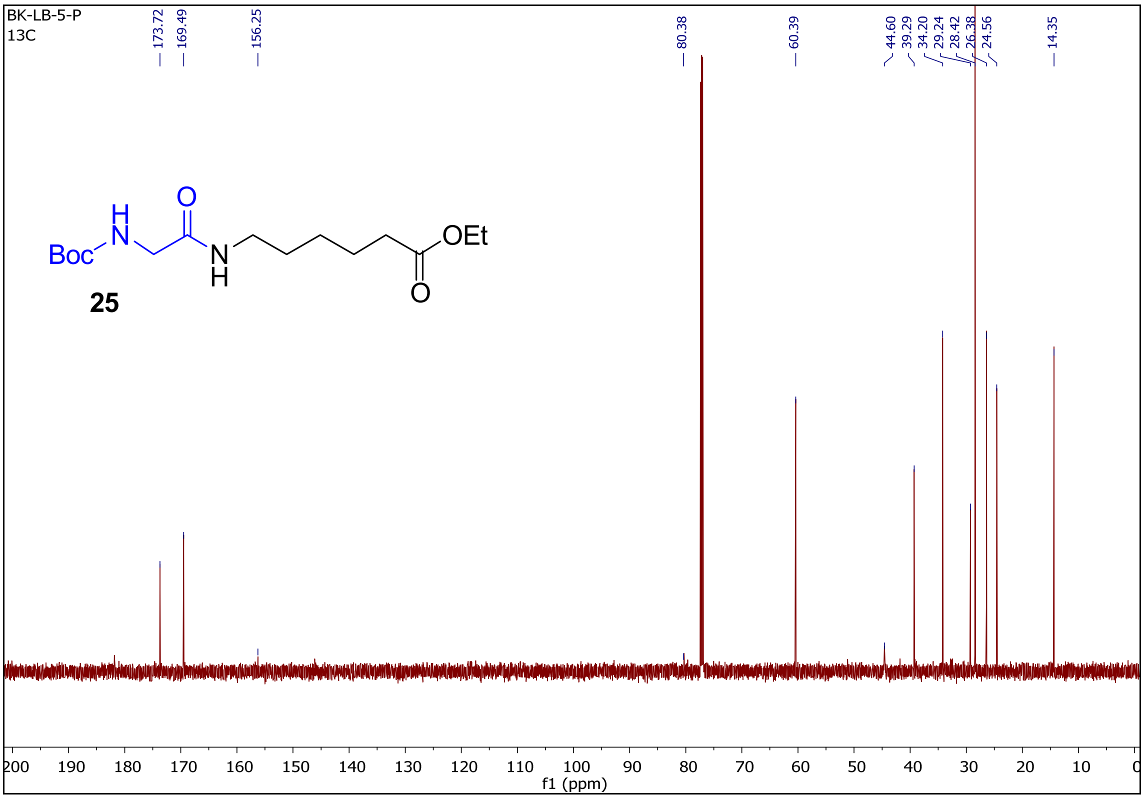

Figure S16. ^{13}C NMR of compound **25** (176 MHz, $CDCl_3$).

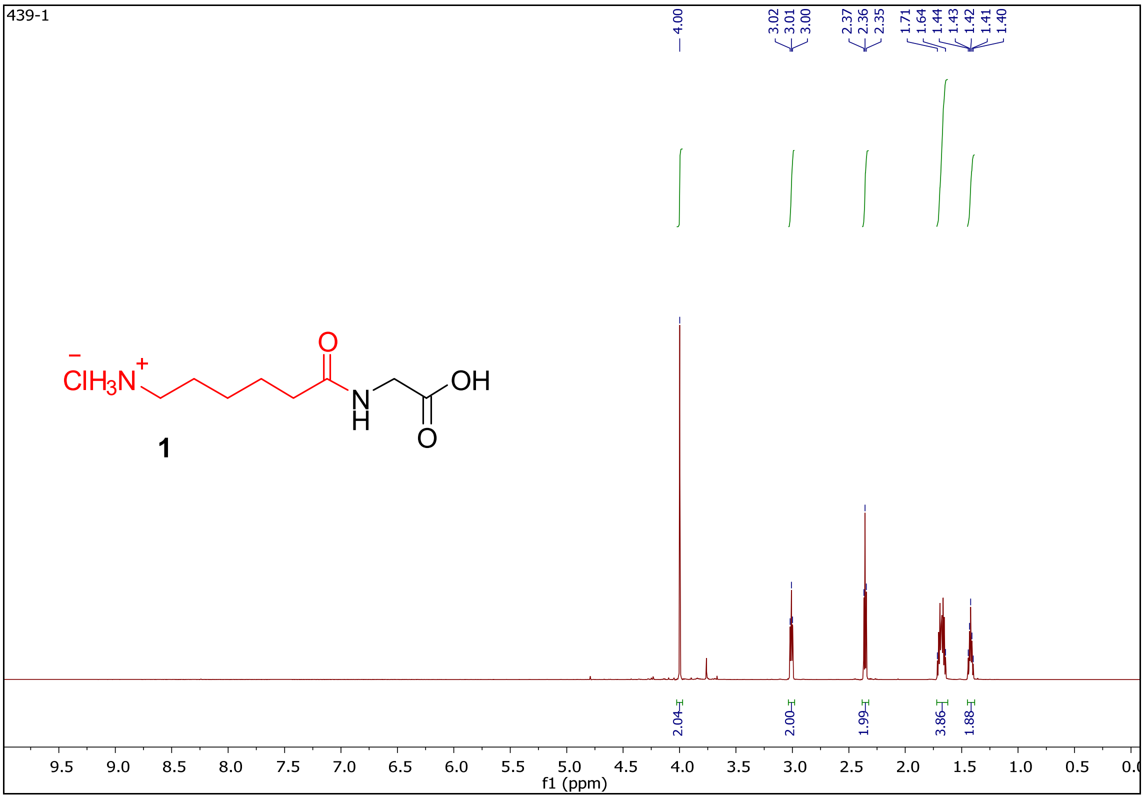

Figure S17. ^{1}H NMR of compound **1** (700 MHz, D_2O).

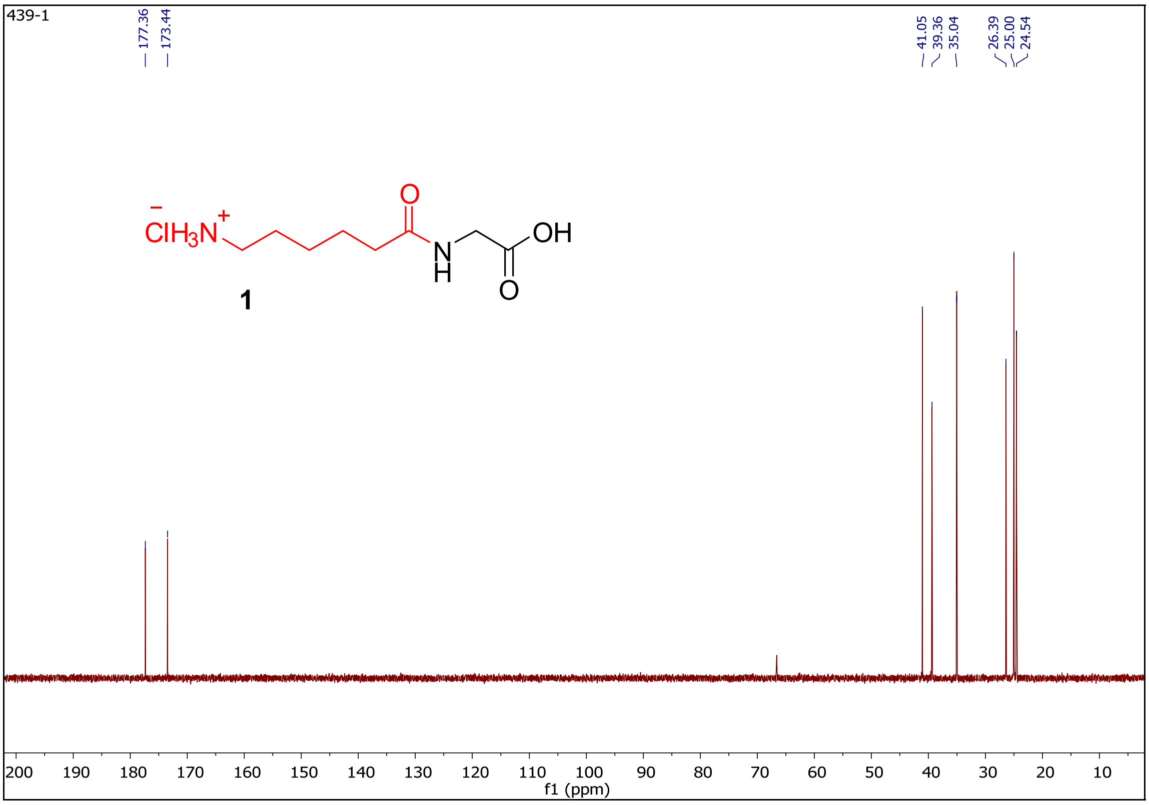

Figure S18. ^{13}C NMR of compound **1** (176 MHz, D_2O).

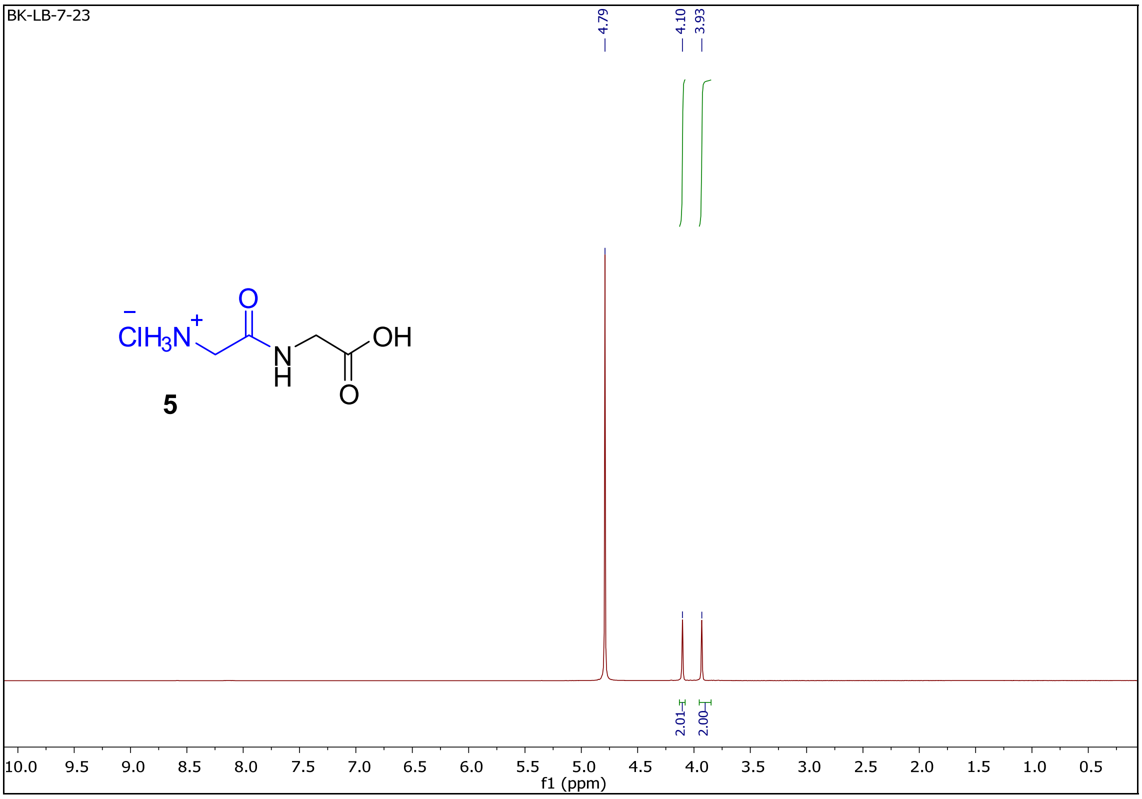

Figure S19. ^{1}H NMR of compound **5** (700 MHz, D_2O).

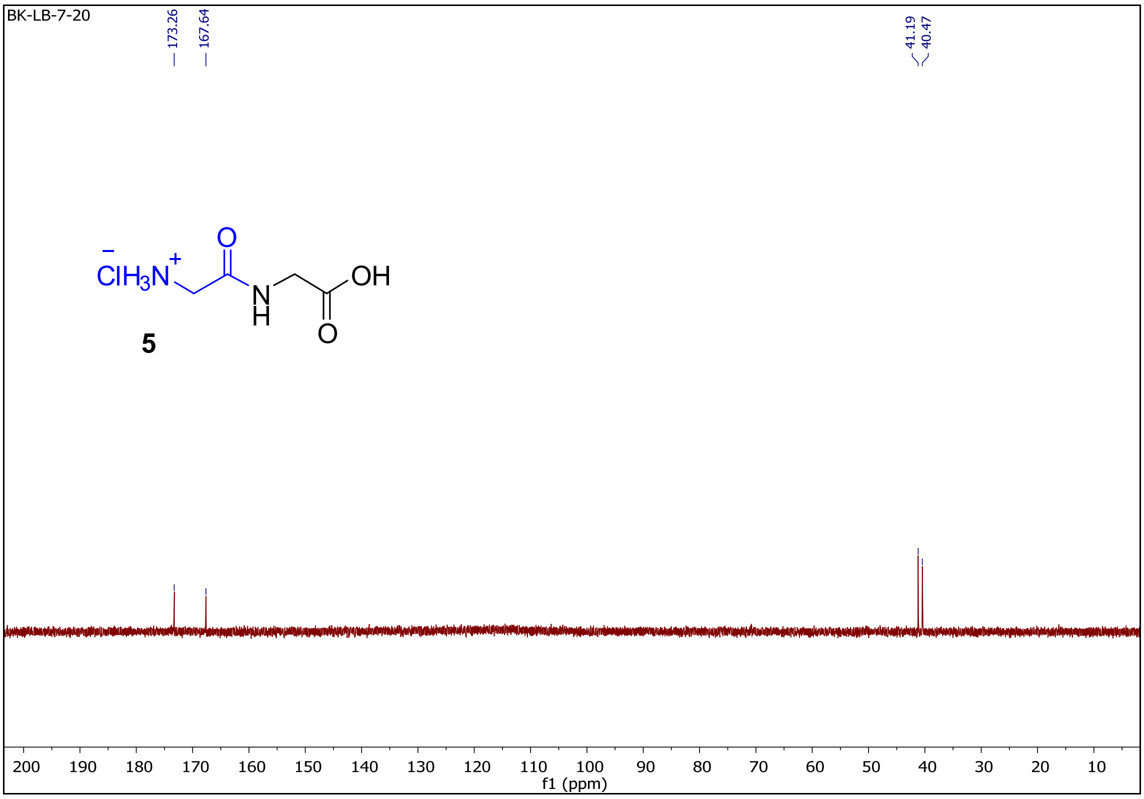

Figure S20. ^{13}C NMR of compound **5** (176 MHz, D_2O).

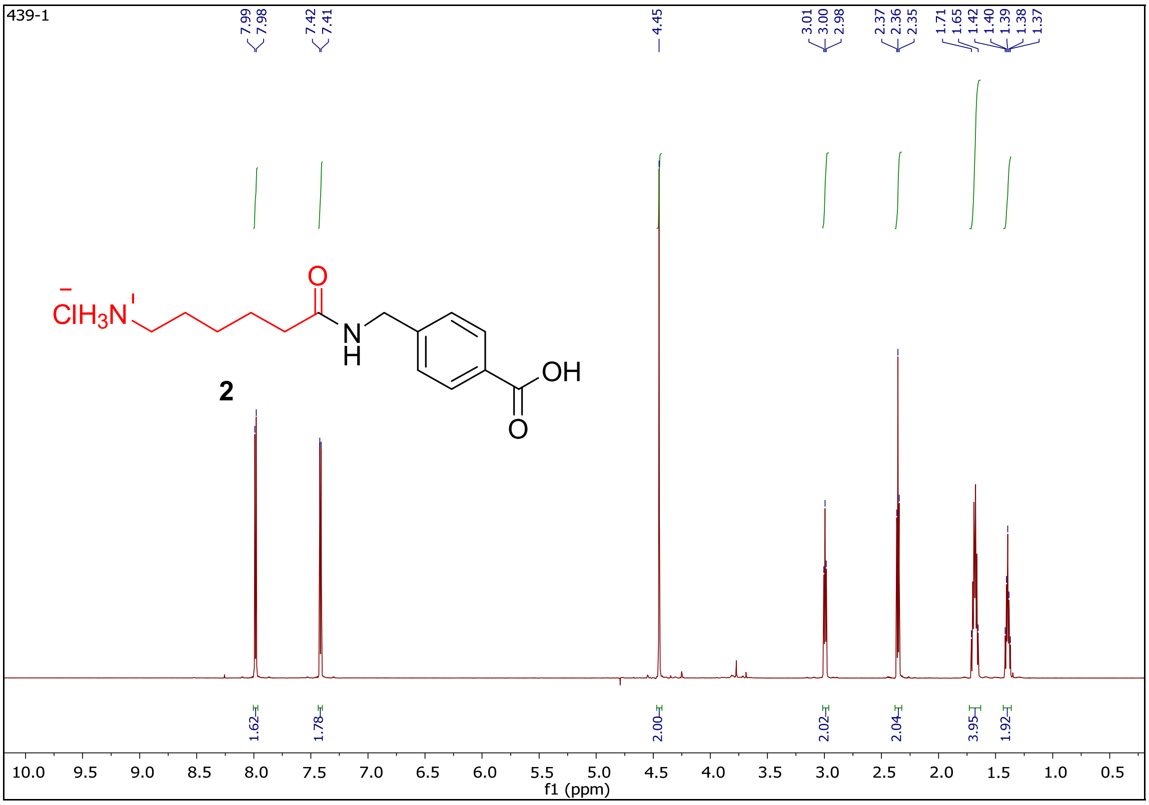

Figure S21. ^{1}H NMR of compound **2**(700 MHz, D_2O).

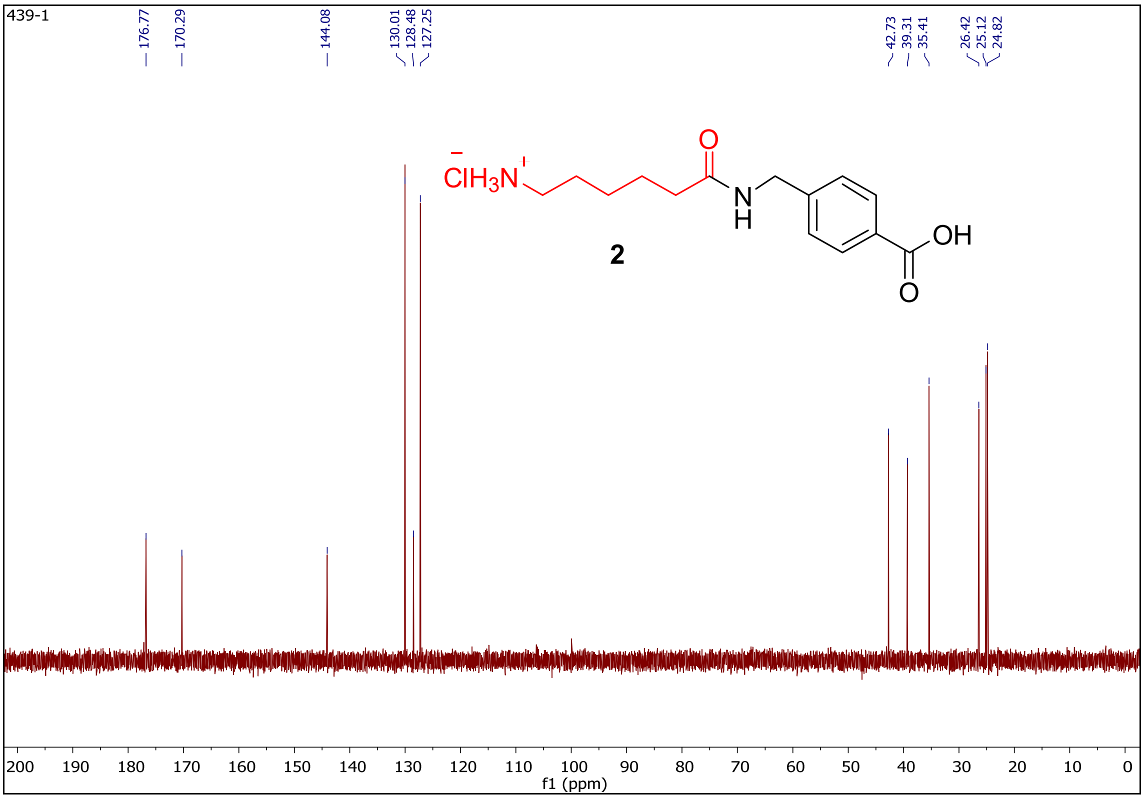

Figure S22. ^{13}C NMR of compound **2** (176 MHz, D_2O).

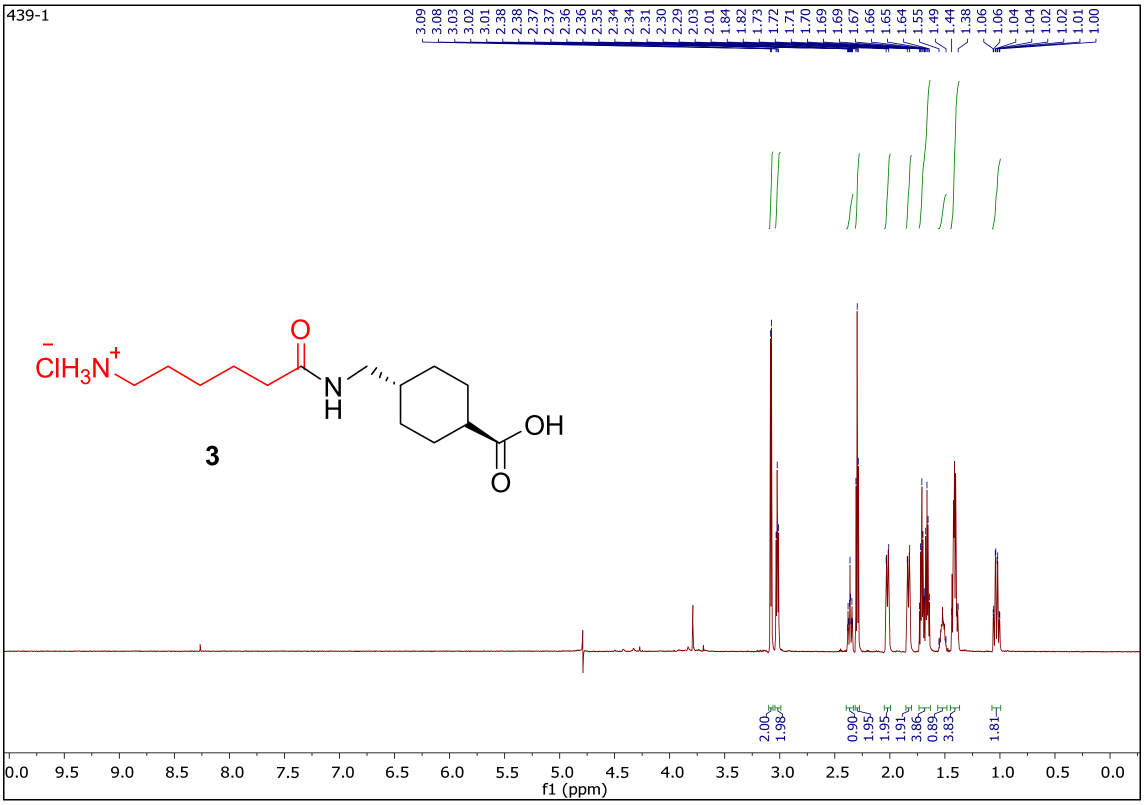

Figure S23. ^1H NMR of compound **3** (700 MHz, D_2O).

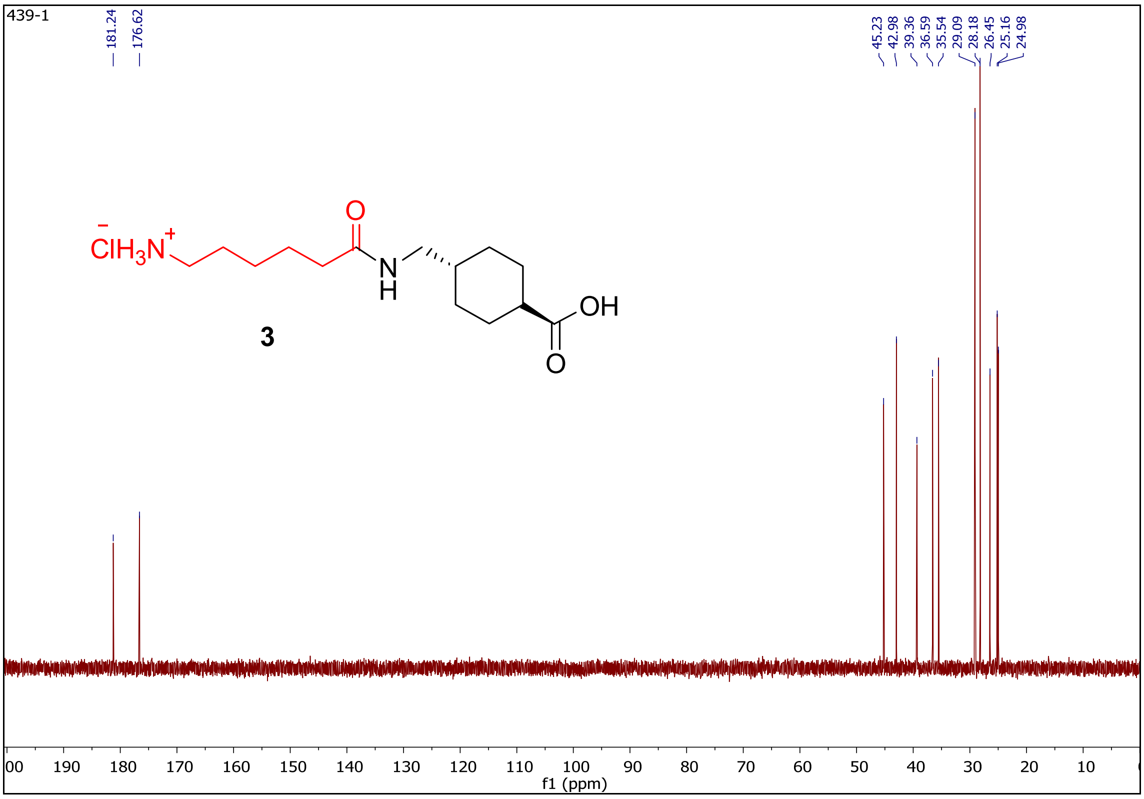

Figure S24. ^{13}C NMR of compound **3** (176 MHz, D_2O).

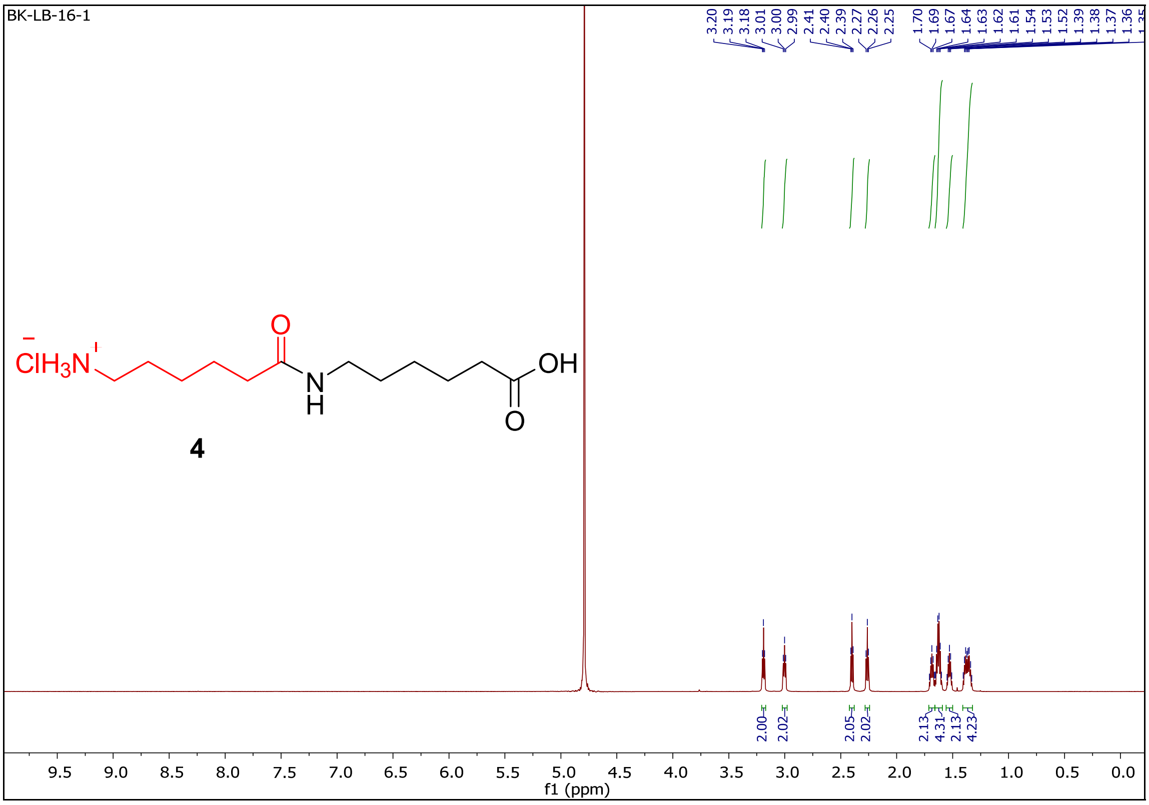

Figure S25. ^{1}H NMR of compound **4** (700 MHz, D_2O).

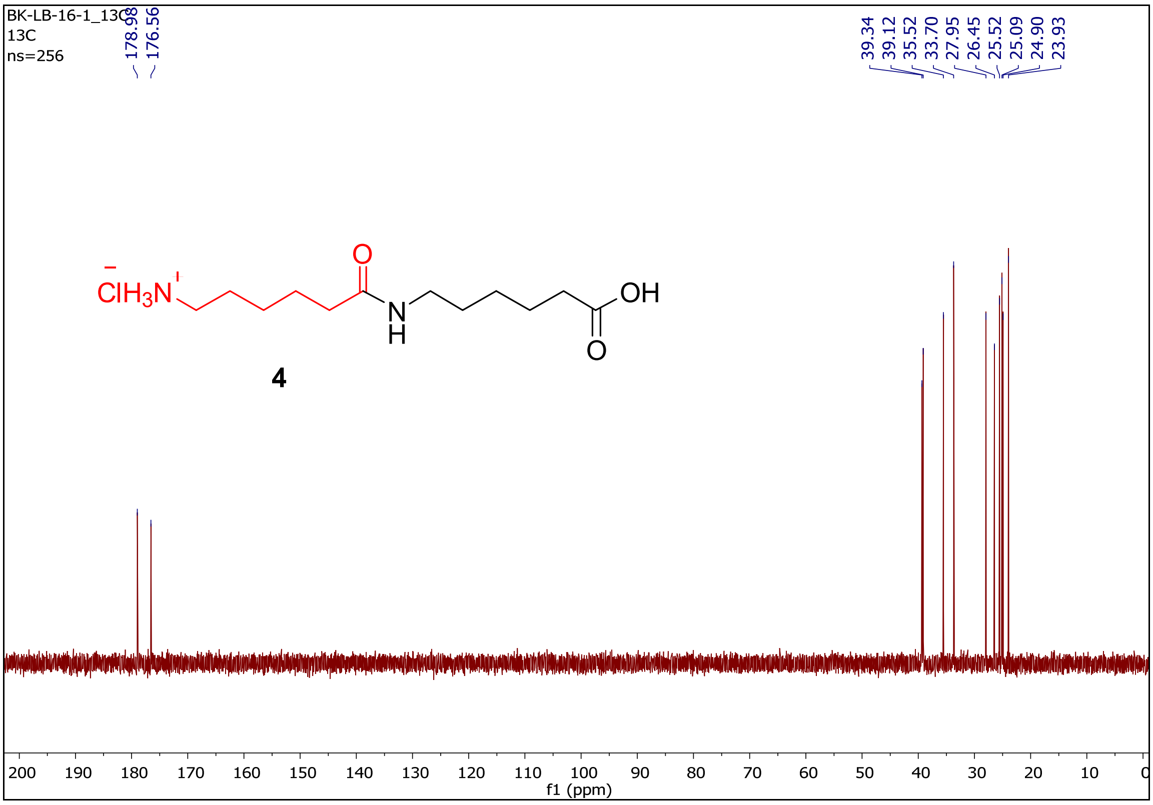

Figure S26. ^{13}C NMR of compound **4** (176 MHz, D_2O).

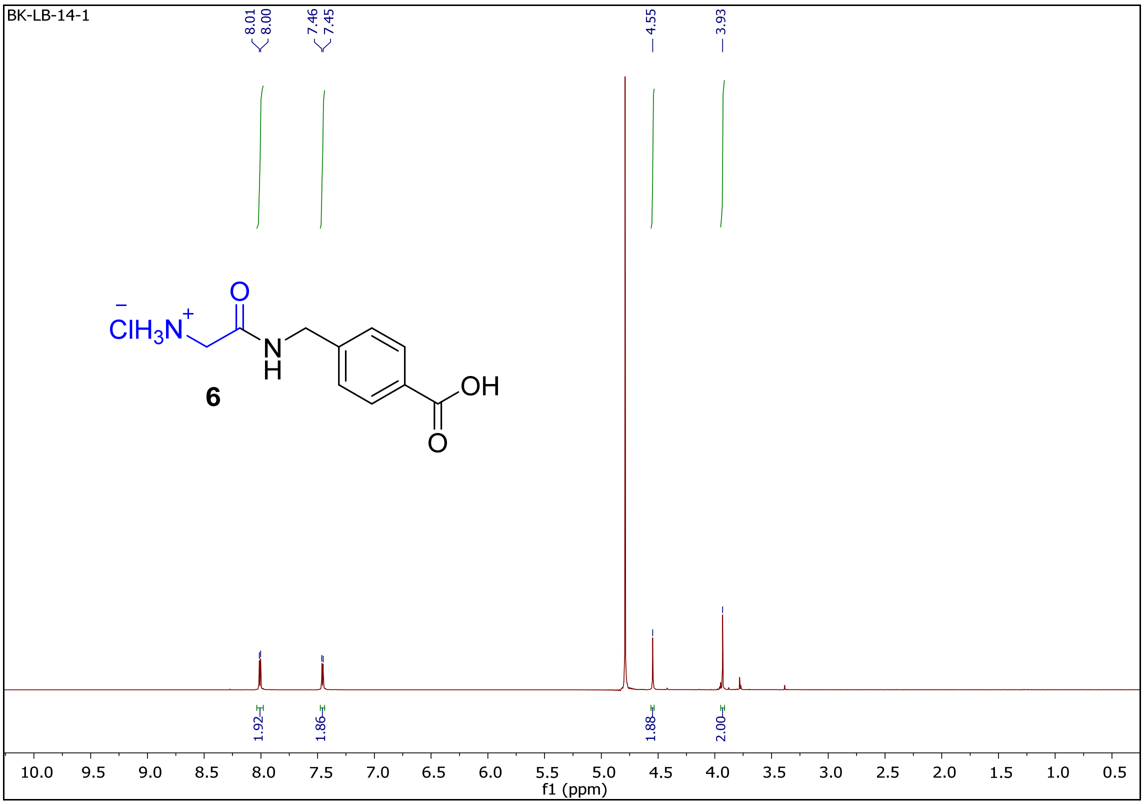

Figure S27. ^{1}H NMR of compound **6** (700 MHz, D_2O).

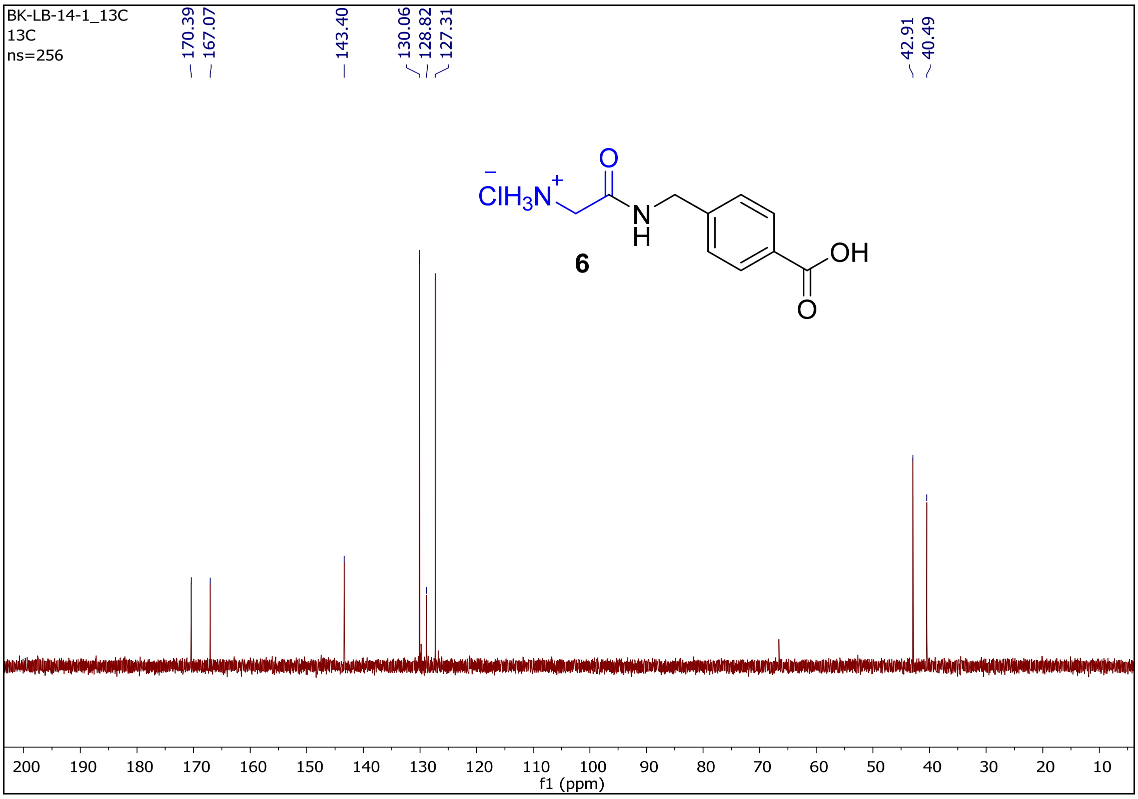

Figure S28. ^{13}C NMR of compound **6** (176 MHz, D_2O).

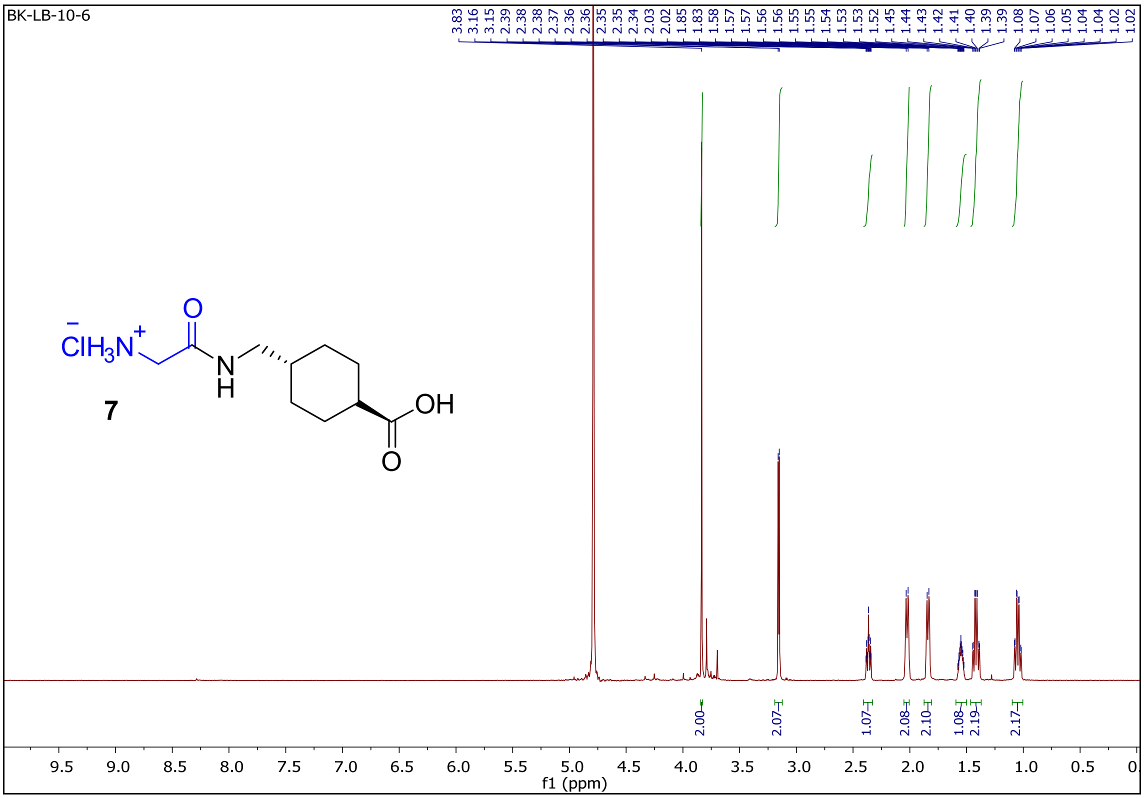

Figure S29. ^{1}H NMR of compound **7** (700 MHz, D_2O).

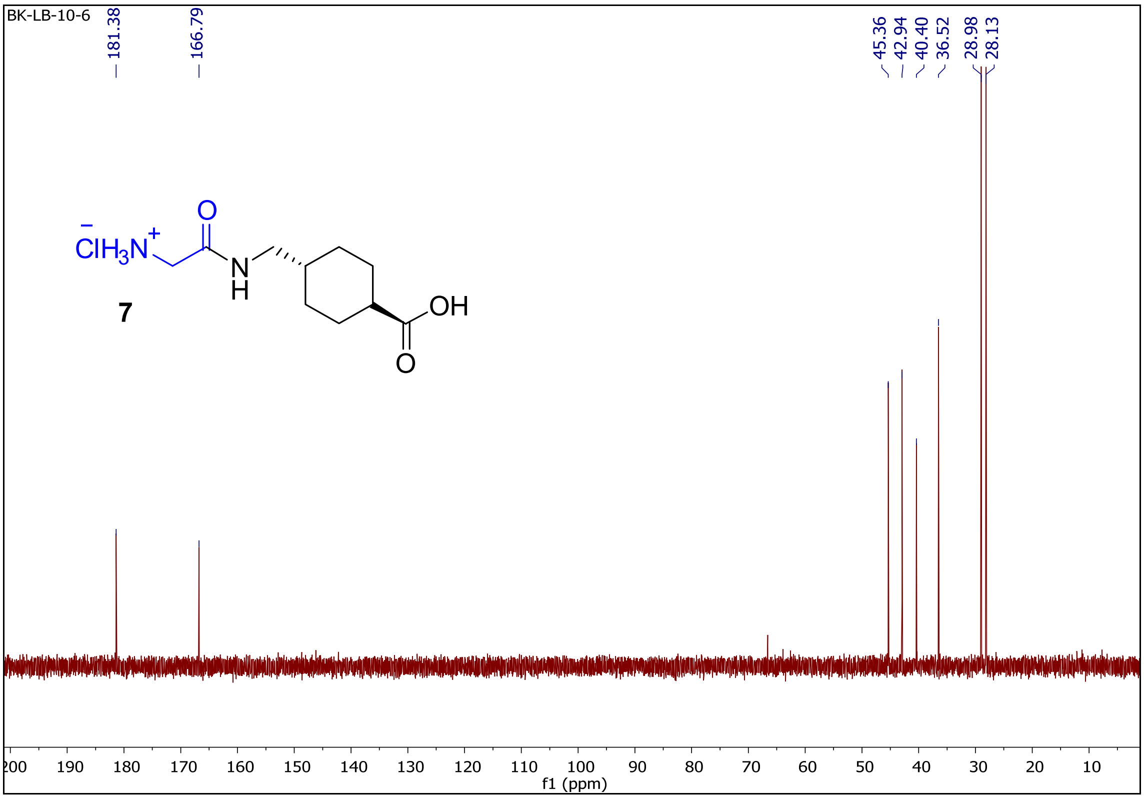

Figure S30. ^{13}C NMR of compound **7** (176 MHz, D_2O).

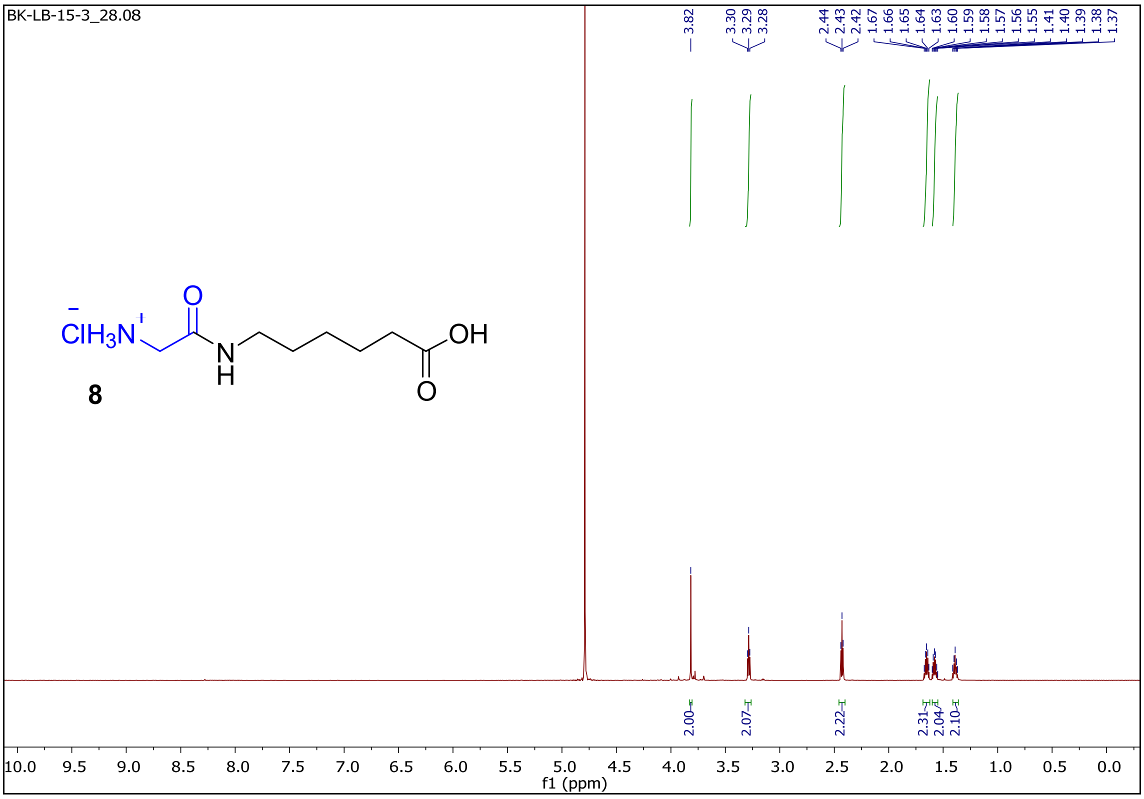

Figure S31. ^{1}H NMR of compound **8** (700 MHz, D_2O).

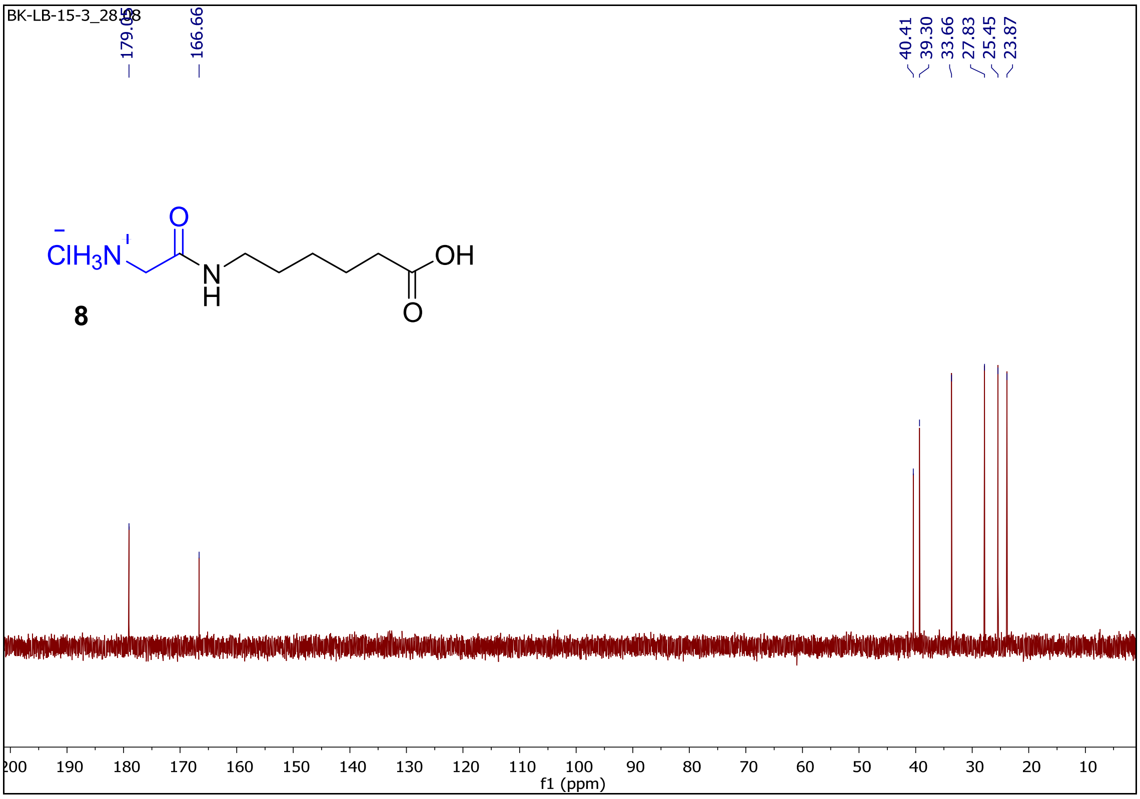

Figure S32. ^{13}C NMR of compound **8** (176 MHz, D_2O).

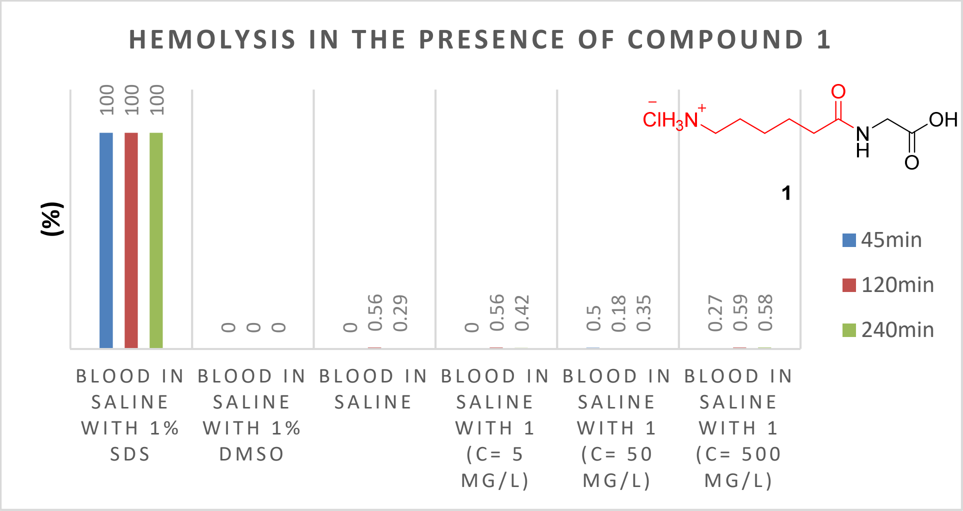

Figure S33. Graph of hemolysis in the presence amide **1**.

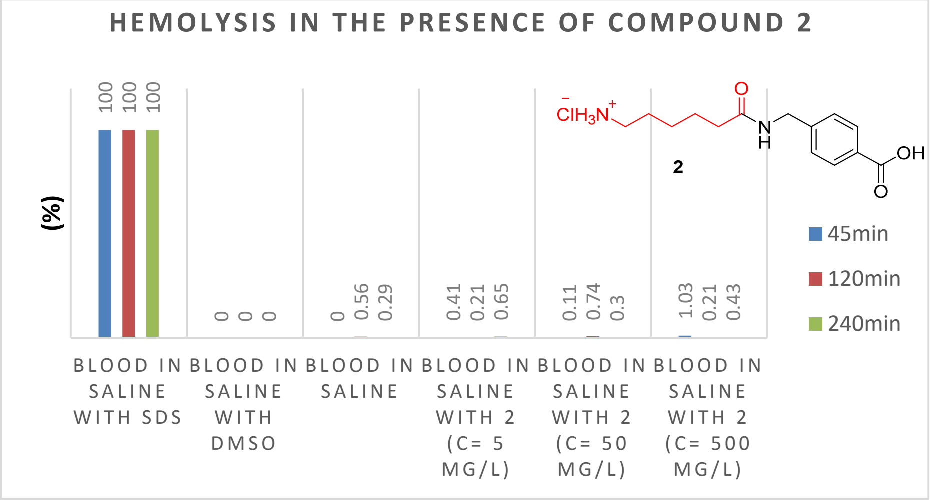

Figure S34. Graph of hemolysis in the presence amide **2**.

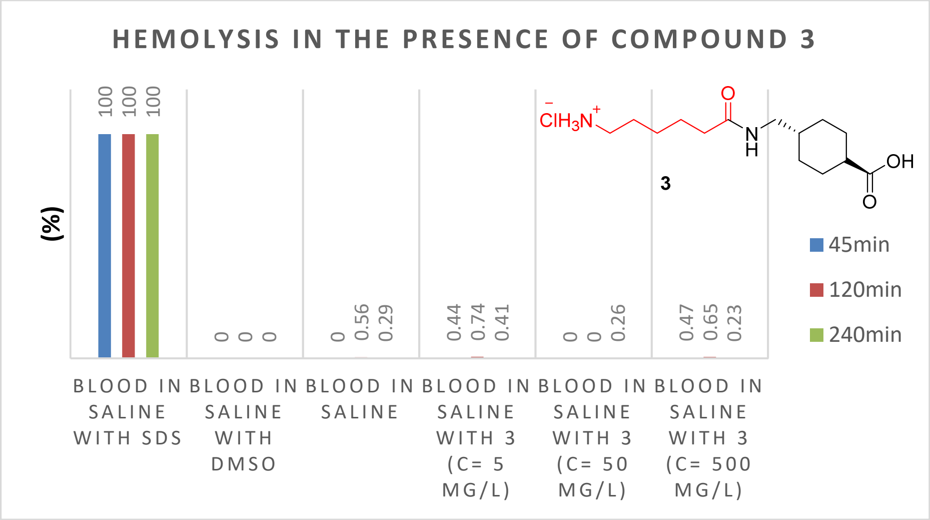

Figure S35. Graph of hemolysis in the presence of amide **3**.

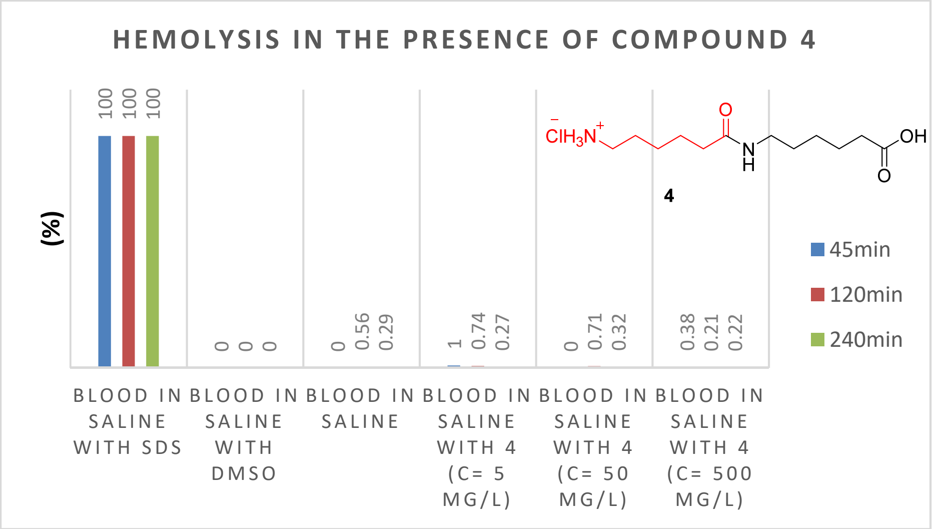

Figure S36. Graph of hemolysis in the presence of amide **4**.

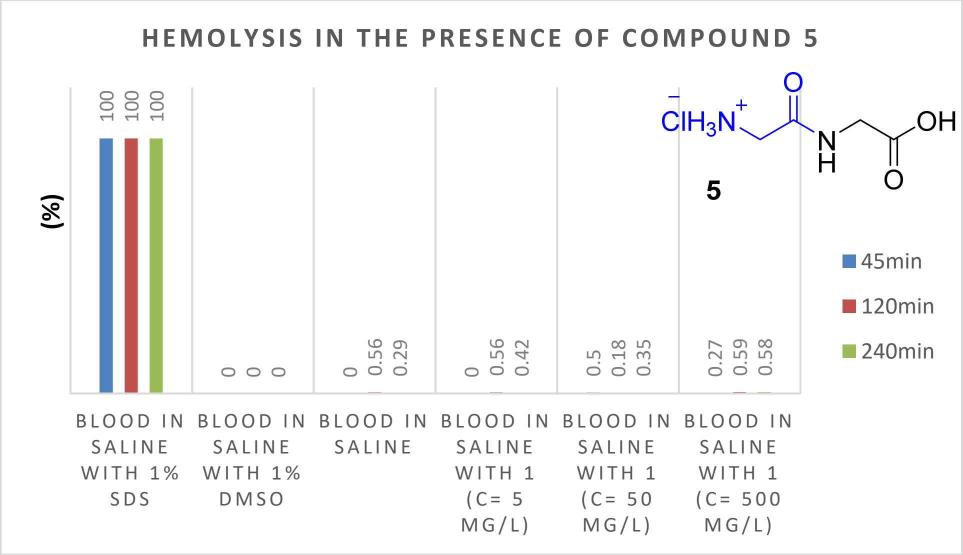

Figure S37. Graph of hemolysis in the presence of amide **5**.

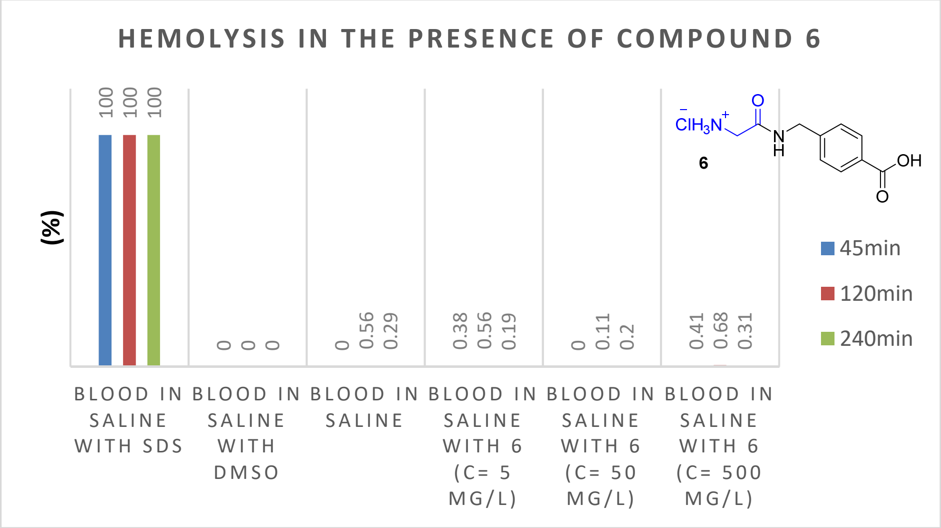

Figure S38. Graph of hemolysis in the presence of **6**.

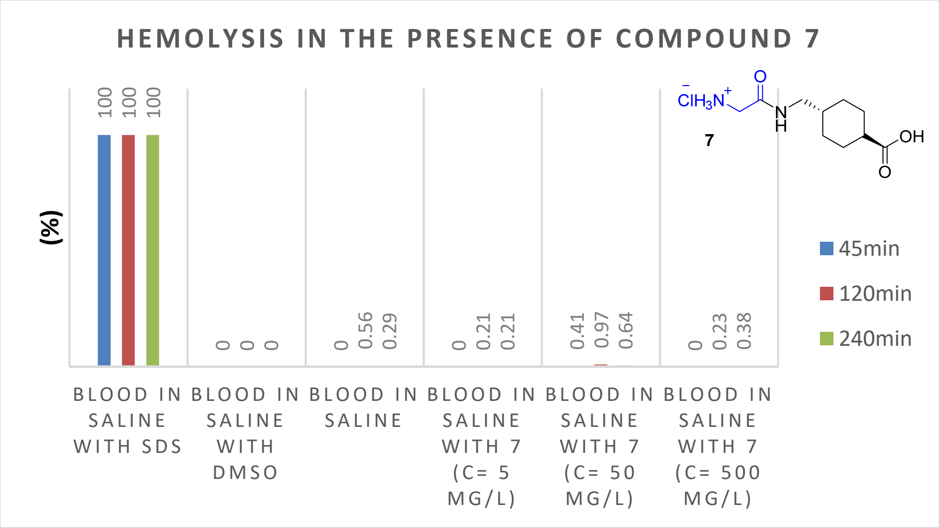

Figure S39. Graph of hemolysis in the presence of amide **7**.

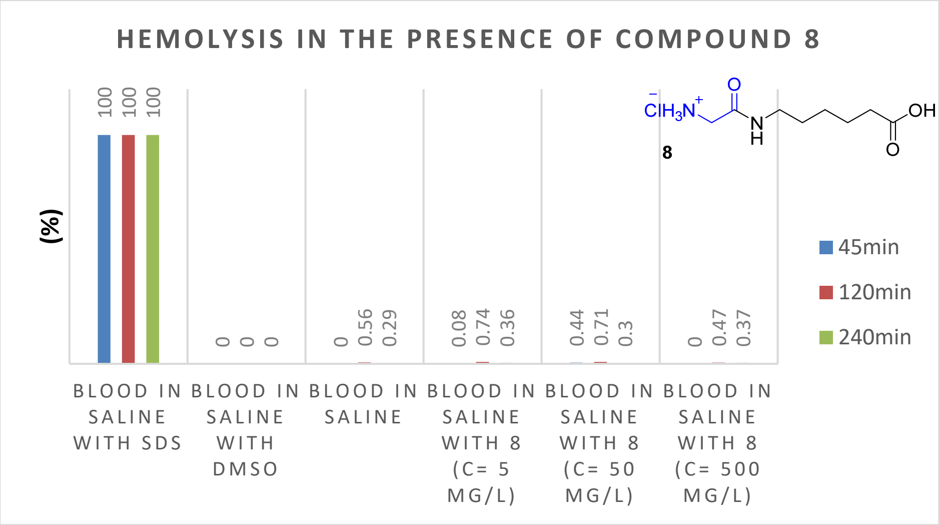

Figure S40. Graph of hemolysis in the presence of amide **8**.

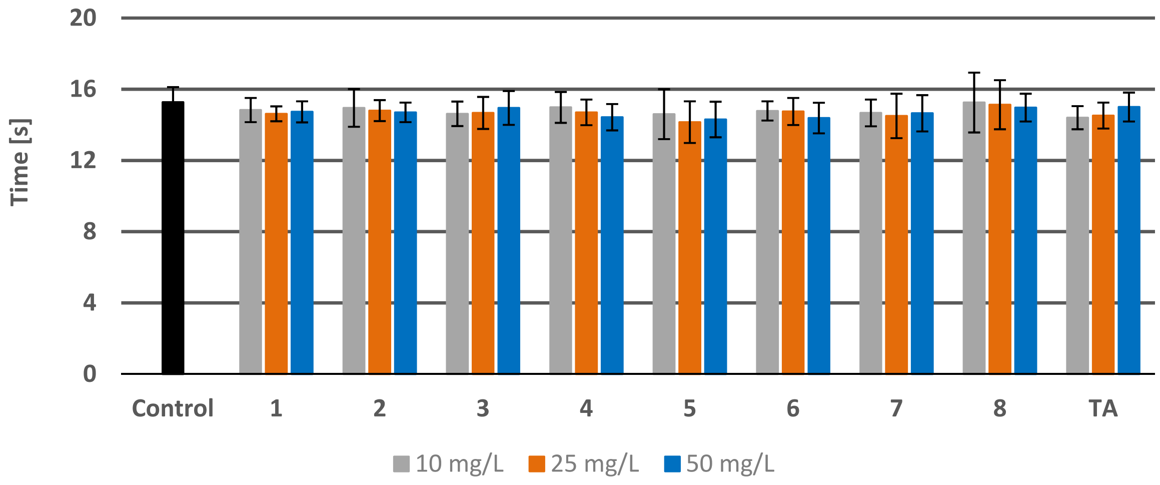

Figure S41. Graph of prothrombin time (PT) in the presence amides **1**–**8** and tranexamic acid (TA) in three different concentrations [10, 25 and 50 mg/L] and for control sample - plasma without any extra reagents (***p <0.05).

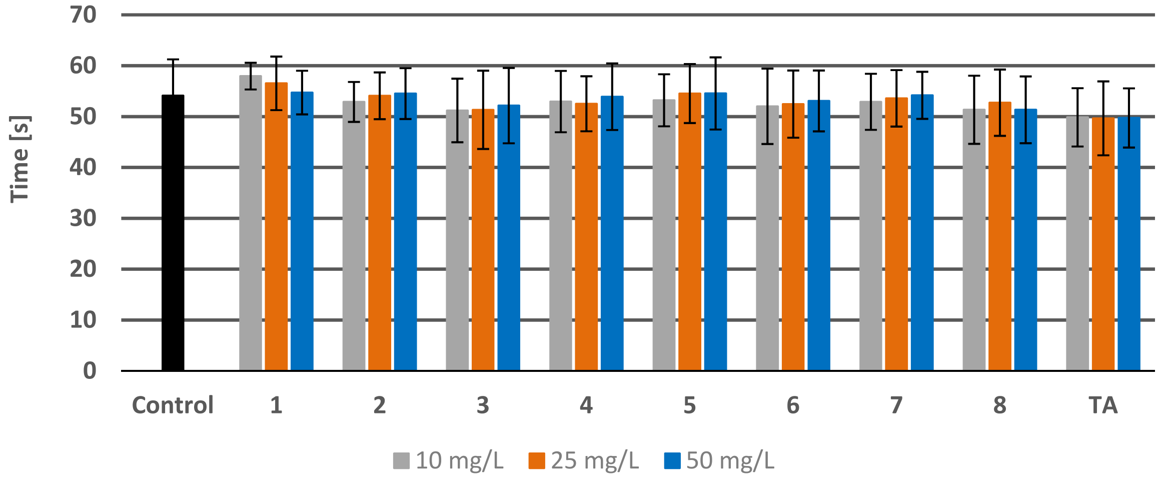

Figure S42. Graph of activated partial thromboplastin time (aPTT) in the presence amides **1**–**8** and tranexamic acid (TA) in three different concentrations [10, 25 and 50 mg/L] and for control sample - plasma without any extra reagents.

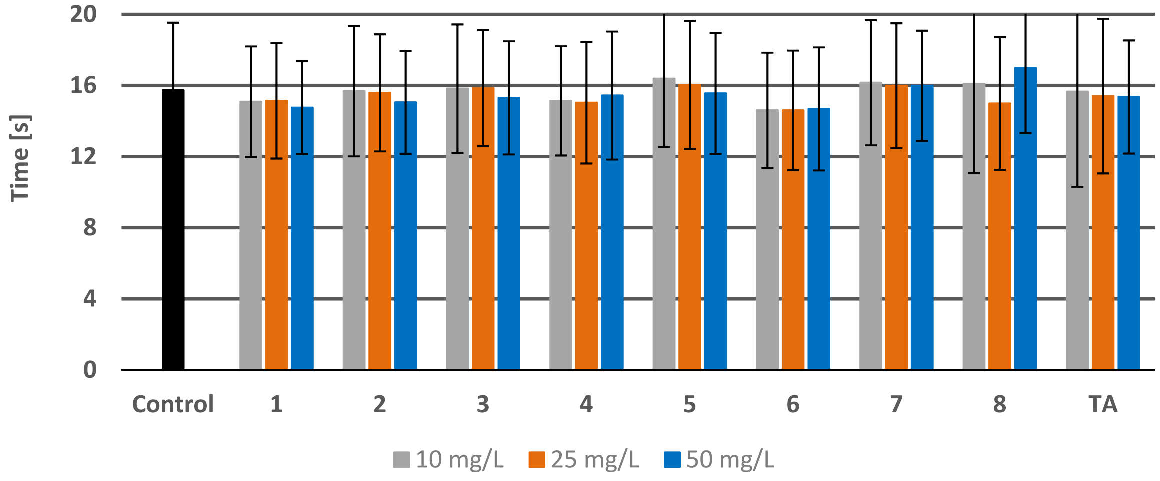

Figure S43. Graph of the thrombin time (TT) in the presence amides **1**–**8** and tranexamic acid (TA) in three different concentrations [10, 25 and 50 mg/L] and for control sample - plasma without any extra reagents.
